# Risk Factors for Synchronous Peritoneal Metastases in Colorectal Cancer: A Systematic Review and Meta-Analysis

**DOI:** 10.3389/fonc.2022.885504

**Published:** 2022-06-20

**Authors:** Yuanxin Zhang, Xiusen Qin, Rui Luo, Hui Wang, Huaiming Wang, Hongzhi Luo

**Affiliations:** ^1^ Department of Colorectal Surgery, The Sixth Affiliated Hospital, Sun Yat-sen University, Guangzhou, China; ^2^ Guangdong Provincial Key Laboratory of Colorectal and Pelvic Floor Disease, The Sixth Affiliated Hospital, Sun Yat-sen University, Guangzhou, China; ^3^ Department of Tumor Surgery, Zhongshan City People’s Hospital, Zhongshan, China

**Keywords:** colorectal cancer, synchronous peritoneal metastases, risk factors, gene, meta-analysis

## Abstract

**Background:**

Early detection of synchronous colorectal peritoneal metastases (CPMs) is difficult due to the absence of typical symptoms and the low accuracy of imaging examinations. Increasing the knowledge of the risk factors for synchronous CPM may be essential for early diagnosis and improving their management. This study aimed to identify the risk factors for synchronous CPM.

**Method:**

The study was registered at PROSPERO (CRD42020198548). The PubMed, Embase and Cochrane Library databases were searched for studies comparing the clinicopathological and molecular features between patients with or without synchronous CPM. The pooled data were assessed by a random-effects model.

**Results:**

Twenty-five studies were included. A synchronous CPM was positively associated with female sex (OR 1.299; 1.118 to 1.509; P = 0.001), PROK1/PROKR2-positivity (OR 2.244; 1.031 to 4.884; P = 0.042), right-sided colon cancer (OR 2.468; 2.050 to 2.970; P < 0.001), poorly differentiated grade (OR 2.560; 1.537 to 4.265; P < 0.001), BRAF mutation (OR 2.586; 1.674 to 3.994; P < 0.001), mucinous adenocarcinoma (OR 3.565; 2.095 to 6.064; P < 0.001), signet-ring cell carcinoma (OR 4.480; 1.836 to 10.933; P = 0.001), N1-2 (OR 5.665; 3.628 to 8.848; P < 0.001), T4 (OR 12.331; 7.734 to 19.660; P < 0.001) and elevated serum CA19-9 (OR 12.868; 5.196 to 31.867; P < 0.001).

**Conclusions:**

These evidence-based risk factors are indicators that could predict the presence of synchronous CPMs and can improve their management.

**Systematic Review Registration:**

www.crd.york.ac.uk/prospero, identifier: CRD42020198548.

## Introduction

Despite the recent improvements in cancer research, colorectal cancer (CRC) has the second highest mortality in both men and women worldwide (1). An important reason for the limited survival in CRC patients is the presence of distant metastasis. In particular, peritoneal metastases (PM) have been shown to be associated with a substantially shorter survival than metastases at other sites (p < 0.001) (2–4). This special type of CRC metastatic disease deserves more attention.

The early detection of synchronous colorectal peritoneal metastasis (CPM) is currently difficult due to the absence of typical symptoms and the low accuracy of noninvasive imaging examinations for nodules smaller than 5 mm (5–7). In fact, a considerable proportion of the cases of synchronous CPM are unexpectedly detected during primary surgery (8). Consequently, if that is the case, the extent of disease can only be evaluated during surgery, and the treatment strategies are often selected at this time, which may cause a suboptimal treatment approach. Many hospitals still lack equipment for hyperthermic intraperitoneal chemotherapy (HIPEC). In addition, the concept and surgical proficiency of cytoreduction surgery may vary among different surgeons (9). These considerations may be unfavourable to the therapeutic strategies for CPMs that are diagnosed during surgery. An improved knowledge of the risk factors for synchronous CPM would increase the level of suspicion of CPMs in patients with no suggestive signs or symptoms and thus could allow physicians to treat these patients more adequately, such as with a more aggressive preoperative examination, with a proactive laparoscopic exploration, or by referring them to specialized centres.

Some studies have been previously conducted in order to identify the risk factors associated with synchronous CPM, but they have had heterogeneous outcomes, such as the location of the primary tumour (2, 10) and MSI-H (11–13). Furthermore, as tumour genotyping has become a standard practice for metastatic colorectal cancer, clinicians now believe that the oncogene mutation status is increasingly clinically relevant, as it may be associated not only with the response to biologic therapies but also with the site-specific metastatic spreading pattern and outcome (14). However, to date, no individualized study that has analysed the molecular features for synchronous CPM has been performed.

Therefore, a comprehensive understanding of the clinicopathological and molecular characteristics of CPM may be necessary for early diagnosis and may help to improve the management of patients who are at high risk of synchronous CPM. A systematic review and meta-analysis of all studies comparing sex, tumour invasion depth, lymph node metastasis, differentiation, location of primary tumour, histological results, and the serum levels of CA19-9, PROK1/PROKR2, BRAF, KRAS, NRAS, PIK3CA and MSI-H/dMMR between synchronous pmCRC and nonpmCRC patients was undertaken.

## Materials and Methods

This systematic review and meta-analysis adhered to the recommendations of the Preferred Reporting Items of Systematic Reviews and Meta-analysis (PRISMA) statement (15). The PRISMA checklist is available in **Supplementary Appendix 1**.

### Study Registration

This study was registered at PROSPERO (International Prospective Register of Systematic Reviews, www.crd.york.ac.uk/prospero). Number CRD42020198548.

### Eligibility Criteria

Referring to the international consensus on colorectal liver metastases (16), synchronous CPM could be defined as peritoneal metastases detected at or before diagnosis or at the time of surgery for the primary CRC.

Comparative studies of primary colorectal tumours (with or without synchronous PM data reported about their clinicopathological and molecular characteristics) were eligible for inclusion. The included studies met the recognized diagnostic criteria as follows: primary colorectal tumours; the primary tumour’s pathological diagnosis was confirmed; and the patient’s synchronous PM was confirmed by an imaging diagnosis before surgery, an intraoperative exploration or by a histopathological examination.

The exclusion criteria were as follows: (1) case reports, review articles and animal studies; (2) non-English publications; (3) studies that were not related to CRC or PM; (4) metachronous PM; (5) no analysis of the risk factors; (6) no comparator group; (7) no relevant data, including articles published only in abstract form as well as studies without complete data or an inability to construct a 2×2 contingency table from the present data; (8) mixed primary tumours; (9) a nonstandardized histological type; and (10) synchronous CPM was not clearly or correctly defined.

### Data Sources and Search Strategy

We selected relevant studies by searching PubMed, Embase and the Cochrane CENTRAL Register of Controlled Trials. The following combined terms were used in the search: (peritoneal metastasis OR peritoneal metastases OR peritoneal carcinomatosis) AND (colorectal OR colon OR rectal). The latest search was implemented on July 14, 2020, and there was not limit to the earliest date of publication.

### Selection Process

Two independent authors (Y Zhang and X Qin) checked the title and abstract of each study, and the studies that satisfied the potential eligibility were obtained for further full-text assessment. Disagreements were resolved by discussion with the senior authors (Huaiming Wang or Hui Wang) until a consensus was achieved.

### Data Extraction

By using standardized forms, two authors (Y Zhang and X Qin) independently extracted the data from each eligible study. The authors resolved any disagreements by discussion with the senior authors (Huaiming Wang or Hui Wang). The following data were extracted from each eligible study: author, year of publication, country where the study was conducted, setting of the centre, type of study, enrolment interval, number of primary CRC patients with or without synchronous PM and the clinicopathological and molecular characteristics. In addition, the Newcastle–Ottawa Scale score (N-O score) was also calculated and extracted for all of the eligible studies.

### Statistical Analysis

We used Comprehensive Meta-Analysis (version 2.0) and Stata (version 12.0) for all statistical analyses. All of the pooled outcomes were determined using a random effects model (DerSimonian–Laird method). In the pooled analyses of the associations between the clinicopathological-molecular characteristics and synchronous PM, the effect sizes were calculated as the odds ratios (ORs) with 95% confidence intervals (CIs). The χ^2^-based Cochran Q test was used to assess heterogeneity among the studies, in which P < 0.1 indicates the presence of heterogeneity (17). We also performed I² inconsistency testing to assess the extent of the heterogeneity among studies, with values greater than 50% regarded as a moderate-to-high heterogeneity (18). For significant heterogeneity, a sensitivity analysis or a subgroup analysis was performed to find the potential source of the heterogeneity. The sensitivity analysis was performed by omitting each study sequentially in order to test the influence of each individual study on the pooled result. The evidence for a publication bias was evaluated by the visual inspection of the funnel plot for symmetry (an asymmetric plot suggested possible publication bias) and was quantified by means of the Begg’s test, with a P value < 0.05 regarded as a significant publication bias (19).

The qualities of the included studies were assessed using the Newcastle–Ottawa Scale (20), in which a score ≥ 6 indicates a high-quality study. The qualities of the studies were evaluated by examining 3 categories: patient selection, comparability of the 2 study groups, and the assessment of exposure (maximum score 9), as shown in the Newcastle–Ottawa Scale.

## Results

### Search and Selection Results

**Figure 1:**
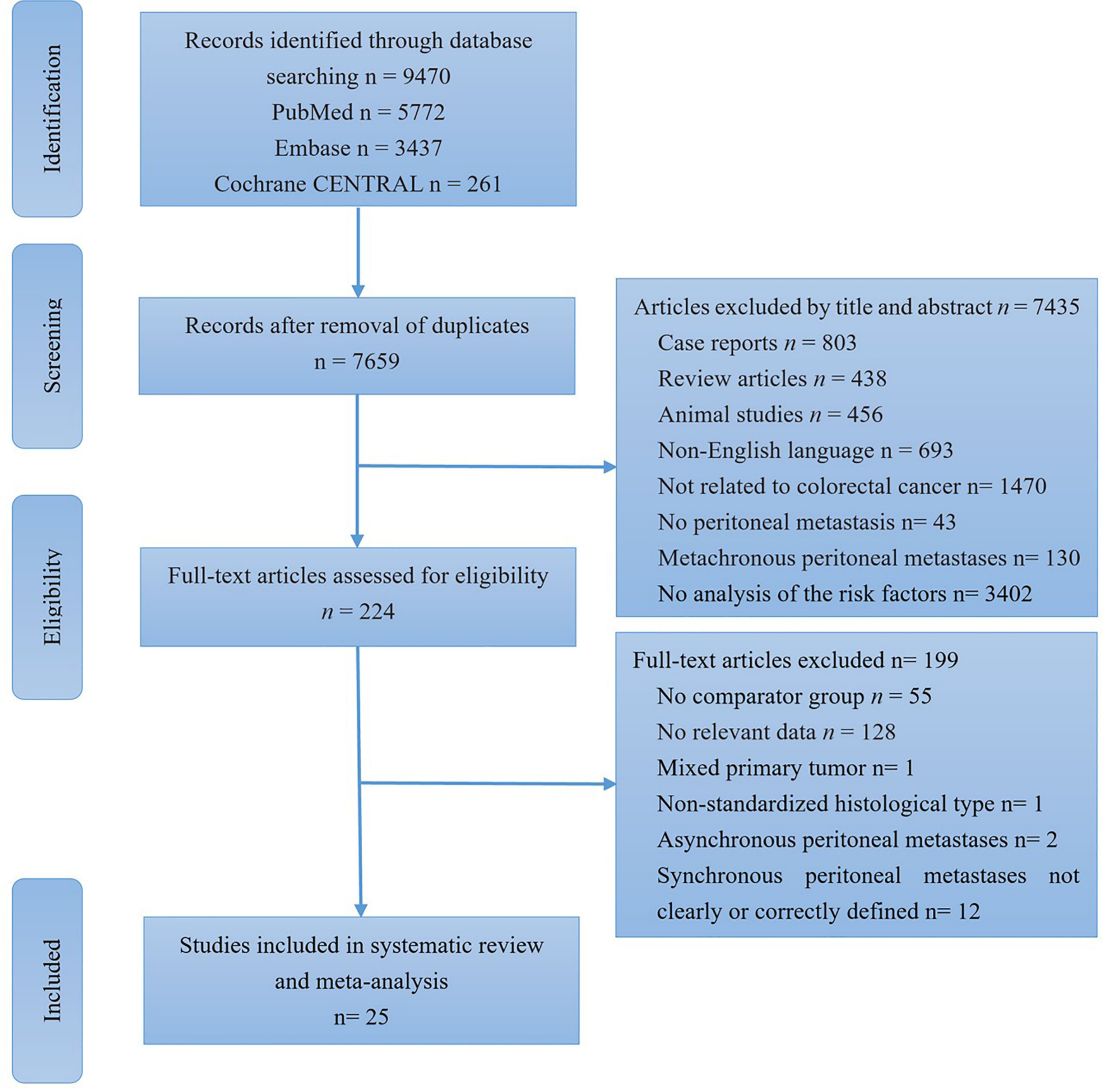
Flow diagram showing the search and selection of studies.

The initial search yielded a total of 9470 studies. After removal of duplicates, a total of 7659 studies were screened by analysing their titles and abstracts, and 7435 studies were removed because they met one or more of the exclusion criteria. The remaining 224 studies were then assessed for eligibility by full-text examination, and a further 199 were excluded due to ineligibility. The reasons for exclusion were recorded. Finally, 25 studies were included in the final analysis (**Figure 1**) (2, 10–13, 21–40).

### Study Characteristics

Among the 25 included studies, 7 had a multicentre setting, and 18 had a single centre design. Five of the included studies were prospectively performed; the remaining twenty were retrospective. All included studies were considered high quality (N-O score ≥ 6). Complete characteristics of the included studies are available in **Table 1**.

**Table 1:** Characteristics of the included studies.

Author	Year	Country	Multicentre/unicentre	Study type	Enrolment interval	Number of patients withsynchronous PM	Number of patients withoutsynchronous PM	Clinical, pathological and biological characteristics	N-Oscore
Sherman et al. (12)	2020	USA	M	Retro	2010-2016	27848	102277	Gender, Differentiation, Histology, KRAS, MSI-H/dMMR	7
Eurboonyanun et al. (24)	2020	USA	U	Retro	2004-2018	17	133	BRAF	8
Cheng et al. (26)	2018	Taiwan	U	Retro	2000-2013	76	260	BRAF	6
Sayagués et al. (25)	2018	Spain	U	Retro	-	7	80	BRAF, KRAS, NRAS, TP53	7
Kaneko et al. (21)	2017	Japan	U	Retro	2009-2015	12	383	Gender, Tumour location, T stage, Differentiation, CA19-9, CEA	8
Jang et al. (28)	2017	Korea	U	Retro	2011-2014	30	319	BRAF, MSI-H/dMMR	8
Sasaki et al. (27)	2017	Japan	U	Retro	2009-2014	13	50	DDR2	6
Franko et al. (2)	2016	ARCAD	M	Pro	1997-2008	1371	9169	Gender, Tumour location, BRAF, KRAS	8
Sasaki et al. (30)	2016	Japan	U	Retro	2006-2011	117	409	Gender, BRAF, KRAS, PIK3CA	8
Huang et al. (29)	2016	Taiwan	U	Retro	2000-2010	14	500	CA125	7
Goi et al. (31)	2015	Japan	U	Retro	1990-2007	9	315	PROK1/PROKR2	7
Cremolini et al. (32)	2015	Italy	M	Retro	2006-2014	138	481	BRAF	6
Shelygin et al. (13)	2014	Russia	U	Retro	2012-2014	20	38	Gender, Tumour location, BRAF, KRAS, MSI-H/dMMR	6
Jimi et al. (33)	2014	Japan	U	Retro	1991-2006	29	397	Histology	8
Nakazawa et al. (34)	2014	Japan	U	Retro	1990-2007	20	600	PROK1/PROKR2	7
Kerscher et al. (22)	2013	Germany	U	Pro	1986-2009	115	2150	Tumour location, T stage, LN+, Histology	8
Smith et al. (11)	2013	UK	M	Pro	2003-2005	36	611	BRAF, KRAS, MSI-H/dMMR, NRAS, PIK3CA	6
Hugen et al. (35)	2013	Netherlands	M	Retro	1991-2010	425	1253	Histology	6
Yu et al. (36)	2013	Korea	U	Pro	2008-2011	12	321	CA19-9	7
Sjo et al. (10)	2011	Norway	M	Pro	1993-2006	94	1030	Gender, Tumour location, T stage, LN+, Histology	8
Lemmens et al. (38)	2011	Netherlands	M	Retro	1995-2008	904	17007	Gender, Tumour location, T stage, LN+, Differentiation, Histology	9
Lin et al. (37)	2011	Taiwan	U	Retro	2001-2003	37	99	CTGF	7
Shirahata et al. (39)	2010	Japan	U	Retro	-	5	39	VIM	6
Song et al. (23)	2009	China	U	Retro	1994-2007	149	1857	Histology	6
Akino et al. (40)	2002	Japan	U	Retro	1986-1999	46	610	Histology, Differentiation	7

M, multicentre; U, unicentre; Retro, retrospective; Pro, prospective; DDR2, discoidin domain receptor 2; PROK1, prokineticin 1; PROKR2, prokineticin receptor 2; CTGF, connective tissue growth factor; VIM, vimentin; LN+, lymph node metastasis.

### Factors not Included in the Quantitative Synthesis

Six clinicopathological and molecular factors could not be included in the quantitative synthesis because they had only a single study in their subgroup, or their methodology did not permit for the pooling of the data. The six factors were serum CEA (21), serum CA125 (29), connective tissue growth factor (CTGF) (37), discoidindomain receptor 2 (DDR2) (27), vimentin (VIM) (39), and TP53 (25). We included these factors in **Table 1** for completeness, but they were not included in the final quantitative synthesis through the meta-analysis.

Finally, 21 studies on 13 factors were included in the quantitative synthesis through the meta-analysis: 7 studies on sex, 4 studies on the tumour invasion depth, 3 studies on lymph node metastasis, 5 studies on the differentiation, 6 studies on the primary tumour site, 7 studies on the histological findings, 2 studies on serum CA19-9, 2 studies on PROK1/PROKR2, 9 studies on BRAF, 6 studies on KRAS, 2 studies on NRAS, 2 studies on PIK3CA and 4 studies on the MSI-H/dMMR status.

### Gender

**Figure 2:**
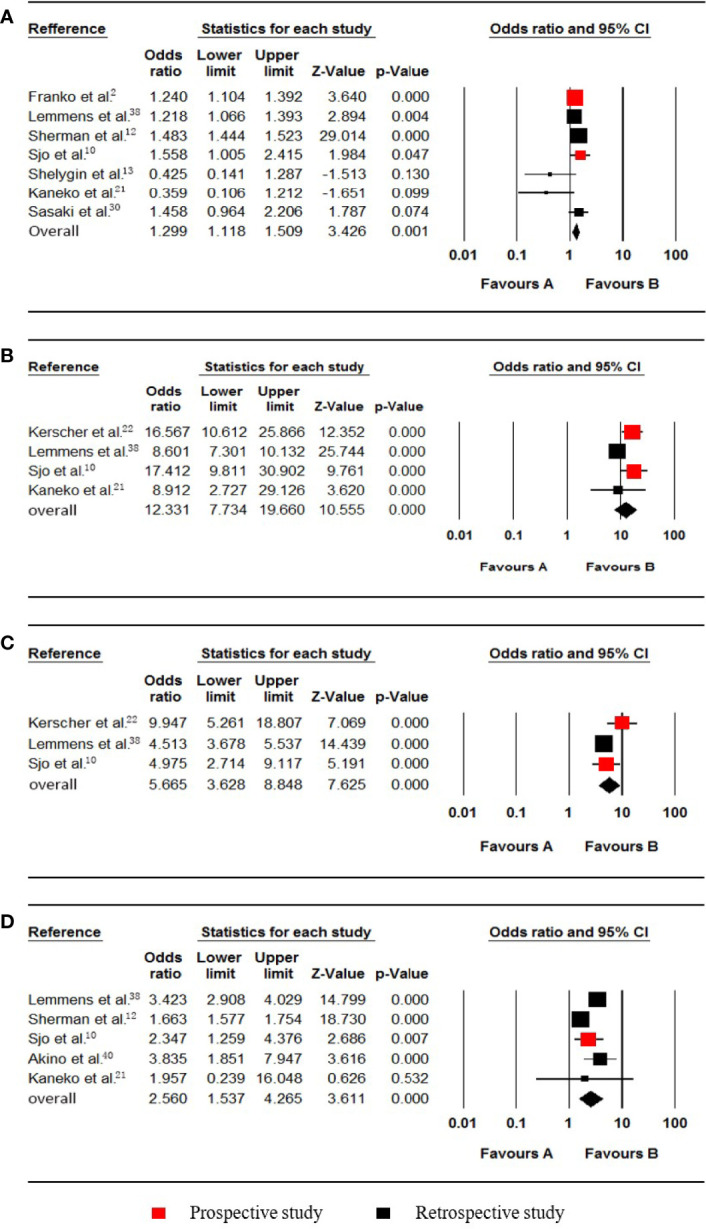
Forest plot for females, T4, N1-2 and poorly differentiated grade. Favours A, non-pmCRC. Favours B, synchronous pmCRC. **(A)** female. **(B)** T4. **(C)** N1-2. **(D)** poorly differentiated grade.

Seven studies (2, 10, 12, 13, 21, 30, 38) that included 160679 patients (30366 synchronous pmCRC, 130313 nonpmCRC) and that evaluated the patients’ sex were included in the meta-analysis. The pooled analysis indicated that females were positively associated with synchronous PM compared to males (OR 1.299; 95% CI, 1.118 to 1.509; P = 0.001) (**Figure 2A**). There was significant heterogeneity (Cochran Q, P < 0.001; I² = 76.9%). To explore the possible sources of the heterogeneity, a sensitivity analysis was performed by omitting each study sequentially to test the influence of each individual study on the pooled result. When one study was omitted (12), there was no significant heterogeneity (Cochran Q, P = 0.099; I² = 46.0%), with no noticeable influence on the pooled OR confidence interval (OR 1.233; 95% CI, 1.051 to 1.445; P = 0.010). It is noteworthy that the proportion of females in the PM group was > 50% in that one study, but in the others, the proportions were < 50%.

### Tumour Invasion Depth

Four studies (10, 21, 22, 38) that included data from 19432 patients (809 synchronous pmCRC, 18623 nonpmCRC) regarding the tumour invasion depth were included in the meta-analysis. The pooled analysis indicated that T4 was positively associated with synchronous PM compared with T1-3 (OR 12.331; 95% CI, 7.734 to 19.660; P < 0.001) (**Figure 2B**). There was significant heterogeneity (Cochran Q, P = 0.009; I² = 74.2%). When one study was omitted (38), there was no significant heterogeneity (Cochran Q, P = 0.593; I² = 0%), with no noticeable influence on the pooled result (OR 16.028; 95% CI, 11.439 to 22.457; P < 0.001).

### Lymph Node Metastasis

Three studies (10, 22, 38) that included data from 16097 patients (702 synchronous pmCRC, 15395 nonpmCRC) and that compared lymph node metastasis were included in the meta-analysis. The pooled analysis indicated that N1-2 was positively associated with synchronous PM compared with N0 (OR 5.665; 95% CI, 3.628 to 8.848; P < 0.001) (**Figure 2C**). There was significant heterogeneity (Cochran Q, P = 0.068; I² = 62.7%). The heterogeneity disappeared if the study was omitted (Cochran Q, P = 0.765; I² = 0%) (22), with no noticeable influence on the pooled result (OR 4.558; 95% CI, 3.755 to 5.533; P < 0.001).

### Differentiation

Five studies (10, 12, 21, 38, 40) that included data on 108360 patients (21986 synchronous pmCRC, 86374 nonpmCRC) and that compared the differentiation, were included in the meta-analysis. The pooled analysis indicated that a poorly differentiated grade was positively associated with synchronous PM compared with a well/moderately differentiated grade (OR 2.560; 95% CI, 1.537 to 4.265; P < 0.001) (**Figure 2D**). There was significant heterogeneity (Cochran Q, P < 0.001; I² = 94.5%). The heterogeneity disappeared when one of the studies was omitted (Cochran Q, P = 0.636; I² = 0%) (12), with no noticeable influence on the pooled result (OR 3.352; 95% CI, 2.875 to 3.909; P < 0.001).

### Location of the Primary Tumour

**Figure 3:**
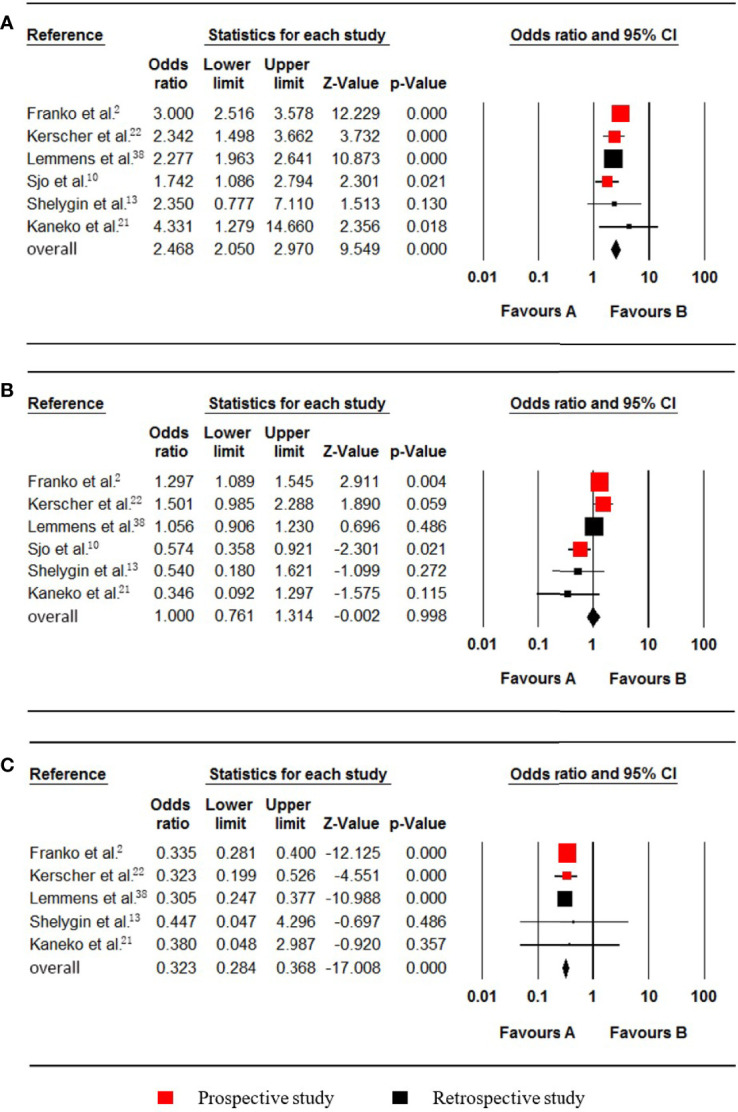
Forest plot for the right colon, left colon and rectum locations. Favours A, non-pmCRC. Favours B, synchronous pmCRC. **(A)** right colon. **(B)** left colon. **(C)** rectum.

Six studies (2, 10, 13, 21, 22, 38) regarding colon cancer were included in the meta-analysis. The PM status of right and left colon cancer patients were listed as follows, respectively: Right colon (720 synchronous pmCRC, 6158 nonpmCRC) and left colon cancer (568 synchronous pmCRC, 7822 nonpmCRC). Quantitative synthesis showed that synchronous PM was positively associated with right colon cancer (OR 2.468; 95% CI, 2.050 to 2.970; P < 0.001) (**Figure 3A**). There was no significant heterogeneity (Cochran Q, P = 0.119; I² = 42.9%). Besides, synchronous PM was not associated with left colon cancer (OR 1.000; 95% CI, 0.761 to 1.314; P = 0.998) (**Figure 3B**). There was significant heterogeneity (Cochran Q, P = 0.004; I² = 71.4%). When one study was omitted through the sensitivity analysis (10), the heterogeneity was less significant (Cochran Q, P = 0.049; I² = 58.0%), with no noticeable influence on the pooled result.

Five studies (2, 13, 21, 22, 38) that included data on 23278 patients (1519 synchronous pmCRC, 21759 nonpmCRC) and that evaluated rectal cancer, were included in the meta-analysis. The pooled analysis indicated that rectal cancer was negatively associated with synchronous PM compared with colon cancer (OR 0.323; 95% CI, 0.284 to 0.368; P < 0.001) (**Figure 3C**). No significant heterogeneity existed (Cochran Q, P = 0.969; I² = 0%).

### Histology

**Figure 4:**
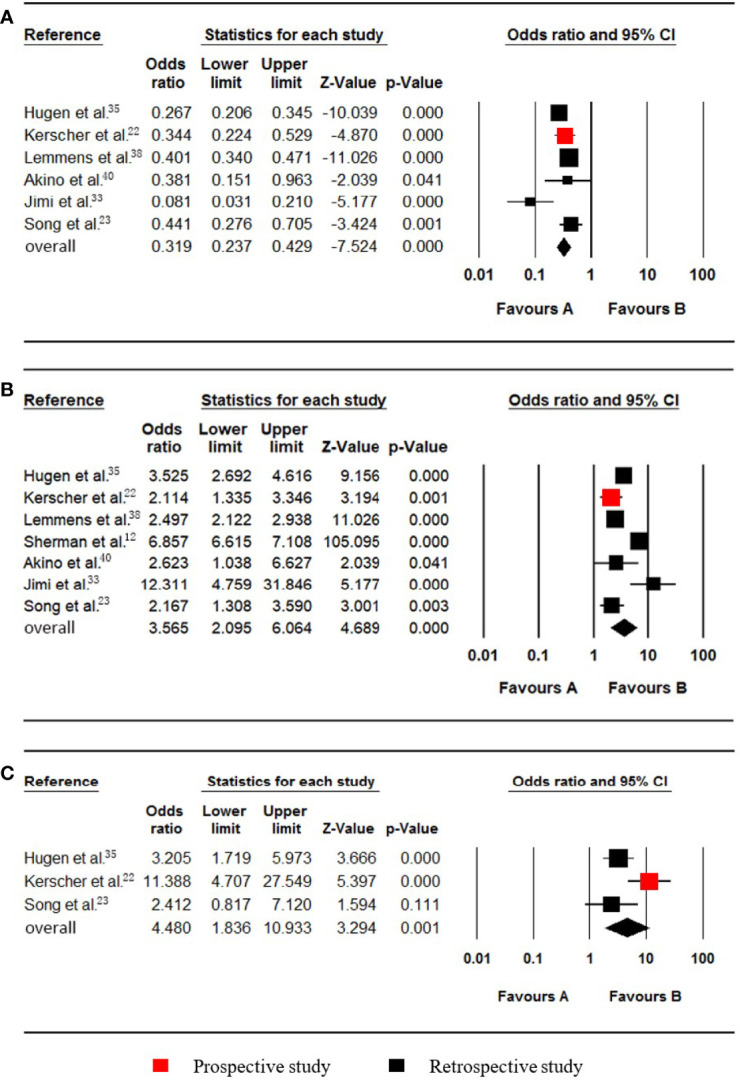
Forest plot for nonmucinous adenocarcinoma (NMC), mucinous adenocarcinoma (MC) and signet-ring cell carcinoma (SRCC). Favours A, non-pmCRC. Favours B, synchronous pmCRC. **(A)** NMC. **(B)** MC. **(C)** SRCC.

Six studies (22, 23, 33, 35, 38, 40), which included data on 24252 patients (1600 synchronous pmCRC, 22652 nonpmCRC) regarding nonmucinous adenocarcinoma (NMC), were included in the meta-analysis. Synchronous PM was negatively associated with NMC (OR 0.319; 95% CI, 0.237 to 0.429; P < 0.001) (**Figure 4A**). There was significant heterogeneity (Cochran Q, P = 0.005; I² = 70.4%). The heterogeneity disappeared if one of the studies was omitted (Cochran Q, P = 0.106; I² = 47.5%) (33), with no noticeable influence on the pooled OR and confidence interval (OR 0.353; 95% CI, 0.285 to 0.437; P < 0.001).

Seven studies (12, 22, 23, 33, 35, 38, 40), which included data on 154377 patients (29448 synchronous pmCRC, 124929 nonpmCRC) regarding mucinous adenocarcinoma (MC), were included in the meta-analysis. Synchronous PM was positively associated with MC (OR 3.565; 95% CI, 2.095 to 6.064; P < 0.001) (**Figure 4B**). There was significant heterogeneity (Cochran Q, P < 0.001; I² = 97.1%). To explore the possible sources of the heterogeneity, a subgroup analysis was performed. According to the rate of PM, two of the studies were divided into subgroup one, and there was no significant heterogeneity (Cochran Q, P = 0.228; I² = 31.2%) (12, 33), with no noticeable influence on the pooled result (OR 7.518; 95% CI, 4.952 to 11.412; P < 0.001). The other studies were divided into subgroup two that also had no significant heterogeneity (Cochran Q, P = 0.174; I² = 37.0%) (22, 23, 35, 38, 40), with no noticeable influence on the pooled result (OR 2.645; 95% CI, 2.169 to 3.226; P < 0.001).

Three studies (22, 23, 35), which included data on 5741 patients (673 synchronous pmCRC, 5068 nonpmCRC) regarding signet-ring cell carcinoma (SRCC), were included in the meta-analysis. Synchronous PM was positively associated with SRCC (OR 4.480; 95% CI, 1.836 to 10.933; P = 0.001) (**Figure 4C**). There was significant heterogeneity (Cochran Q, P = 0.036; I² = 69.7%). When one study was omitted (22), there was no significant heterogeneity (Cochran Q, P = 0.656; I² = 0%) and no noticeable influence on the pooled result (OR 2.986; 95% CI, 1.741 to 5.123; P < 0.001). It is noteworthy that the omitted study had a much higher OR value.

### Serum CA19-9

**Figure 5:**
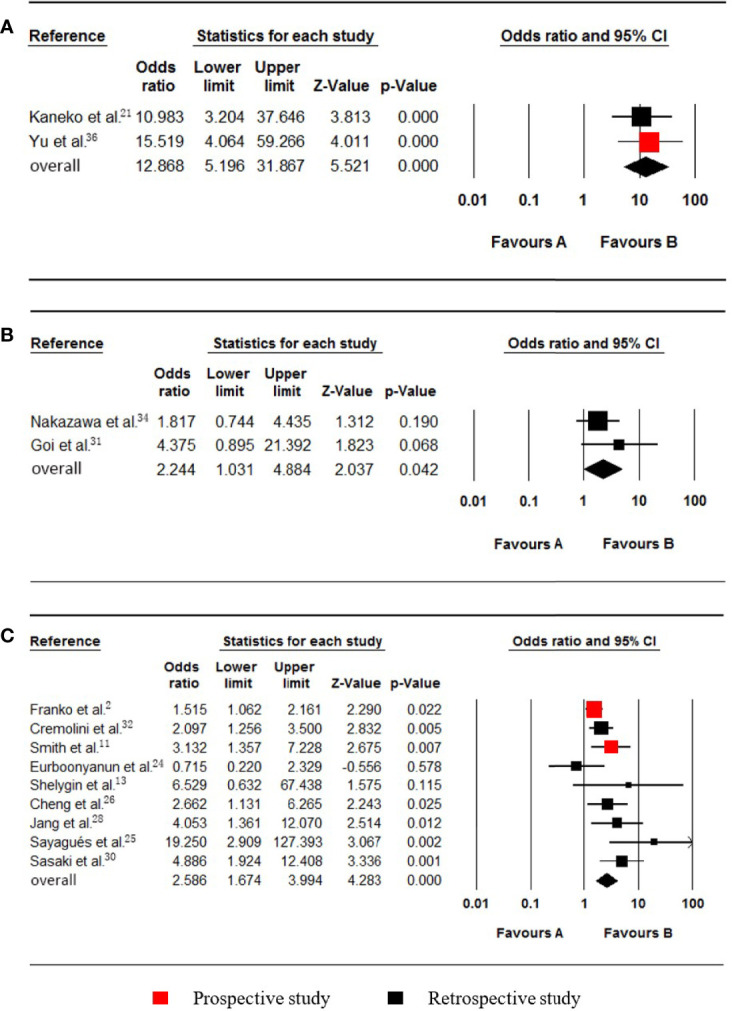
Forest plot for serum CA19-9, PROK1/PROKR2 and BRAF. Favours A, non-pmCRC. Favours B, synchronous pmCRC. **(A)** serum CA19-9. **(B)** PROK1/PROKR2. **(C)** BRAF.

Levels of up to 37.0 µ/ml were taken as the upper cut-off values for the Serum CA19-9 reference ranges. Two studies (21, 36), which included data on 728 patients (24 synchronous pmCRC, 704 nonpmCRC) regarding serum CA19-9, were included in the meta-analysis. Synchronous PM was positively associated with elevated serum CA19-9 (OR 12.868; 95% CI, 5.196 to 31.867; P < 0.001) (**Figure 5A**). No significant heterogeneity existed (Cochran Q, P = 0.710; I² = 0%).

### PROK1/PROKR2

Two studies (31, 34), which included data on 944 patients (29 synchronous pmCRC, 915 nonpmCRC) regarding PROK1/PROKR2, were included in the meta-analysis. Synchronous PM was positively associated with PROK1/PROKR2 positivity (OR 2.244; 95% CI, 1.031 to 4.884; P = 0.042) (**Figure 5B**). There was no significant heterogeneity (Cochran Q, P = 0.344; I² = 0%).

### BRAF Status

Nine studies (2, 11, 13, 24–26, 28, 30, 32) that included data on 4979 patients (704 synchronous pmCRC, 4275 nonpmCRC) regarding the patients’ BRAF statuses, were included in the meta-analysis. Synchronous PM was positively associated with BRAF mutations (OR 2.586; 95% CI, 1.674 to 3.994; P < 0.001) (**Figure 5C**). There was significant heterogeneity (Cochran Q, P = 0.019; I² = 56.3%). When one study was omitted (25), there was no significant heterogeneity (Cochran Q, P = 0.073; I² = 45.9%) and no noticeable influence on the pooled result (OR 2.305; 95% CI, 1.569 to 3.385; P < 0.001). It is clear that the study had a smaller sample size.

### KRAS Status

**Figure 6:**
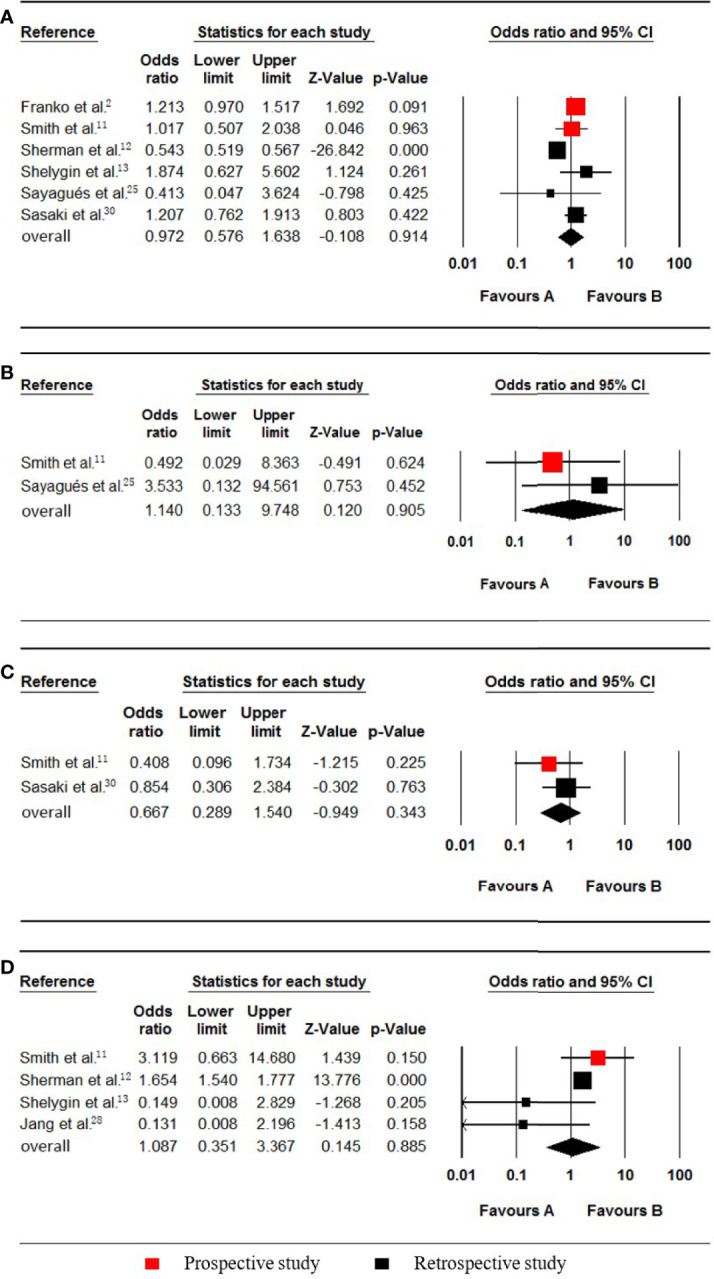
Forest plot for KRAS, NRAS, PIK3CA and MSI-H/dMMR. Favours A, non-pmCRC. Favours B, synchronous pmCRC. **(A)** KRAS. **(B)**, NRAS. **(C)** PIK3CA. **(D)**, MSI-H/dMMR.

Six studies (2, 11–13, 25, 30), which included data on 134197 patients (28362 synchronous pmCRC, 105835 nonpmCRC) regarding the KRAS status, were included in the meta-analysis. Synchronous PM was not associated with KRAS mutations (OR 0.972; 95% CI, 0.576 to 1.638; P = 0.914) (**Figure 6A**). There was significant heterogeneity (Cochran Q, P < 0.001; I² = 92.4%). The heterogeneity disappeared if one of the studies was omitted (Cochran Q, P = 0.774; I² = 0%) [12], with no noticeable influence on the pooled result (OR 1.202; 95% CI, 0.994 to 1.453; P = 0.057). It was found that the rate of KRAS mutations in the synchronous PM group was much lower in the omitted study.

### NRAS Status

Two studies (11, 25), which included data on 731 patients (43 synchronous pmCRC, 688 nonpmCRC) regarding the patients’ NRAS status, were included in the meta-analysis. Synchronous PM was not associated with NRAS mutations (OR 1.140; 95% CI, 0.133 to 9.748; P = 0.905) (**Figure 6B**). No significant heterogeneity existed (Cochran Q, P = 0.373; I² = 0%).

### PIK3CA Status

Two studies (11, 30), which included data on 897 patients (93 synchronous pmCRC, 804 nonpmCRC) regarding the PIK3CA status, were included for eligibility in the meta-analysis. Synchronous CPM was not associated with PIK3CA mutations (OR 0.667; 95% CI, 0.289 to 1.540; P = 0.343) (**Figure 6C**). There was no significant heterogeneity (Cochran Q, P = 0.415; I² = 0%).

### MSI-H/dMMR Status

Four studies (11–13, 28), which included data on 131015 patients (27922 synchronous pmCRC, 103093 nonpmCRC) regarding their MSI-H/dMMR status, were included in the meta-analysis. Synchronous CPM was not associated with MSI-H/dMMR (OR 1.087; 95% CI, 0.351 to 3.367; P = 0.885) (**Figure 6D**). There was significant heterogeneity (Cochran Q, P = 0.097; I² = 52.5%). When one study was omitted (13), there was no significant heterogeneity (Cochran Q, P = 0.153; I² = 46.6%), with no noticeable influence on the pooled result (OR 1.481; 95% CI, 0.536 to 4.087; P = 0.449).

### Publication Bias

No significant publication bias was found according to the visual inspection of the funnel plot and Begg’s test (**Supplementary Figures S1**–**S6**).

## Discussion

This study has provided an extensive analysis for the association between synchronous CPM and its clinicopathological-molecular features. We found that synchronous CPM was positively associated with female sex, PROK1/PROKR2 positivity, a right-sided colon cancer location, a poorly differentiated grade, BRAF mutations, mucinous adenocarcinoma, signet-ring cell carcinoma, N1-2, T4 and an elevated serum CA19-9 (ascendingly sequenced by value of the odds ratios). However, synchronous CPM was not associated with KRAS, NRAS, or PIK3CA mutations or MSI-H/dMMR.

Some studies have previously defined the degree of risk of developing colorectal peritoneal carcinomatosis (41, 42). A high risk of synchronous CPM should modify the management strategy for this special type of metastatic disease, and the following suggestions are given (9, 41). First, in the CRC patients who are at high risk of developing synchronous PM, a more aggressive preoperative examination, such as including PET-CT and diffusion-weighted MRI in the preoperative examination, is suggested to confirm whether there is synchronous PM. Then, if PM is suspected on the preoperative imaging, we propose performing a laparoscopic exploration of the abdominal cavity to assess the extent of the disease and to obtain histological confirmation. Eventually, if synchronous PM is diagnosed, surgeons are expected to describe the extent of the disease and to determine whether aggressive treatment, including complete CRS plus HIPEC, should be given to the patients.

Based on the hypothesis that phenotype and the subsequent clinical behaviour of CPM are driven by underlying biological mechanisms, studies that investigate disease biology will contribute to more precise identification of the suitable patients and for the guidance of therapy. This is one of the critical future research targets in CPM research. The potential mechanisms of the risk factors that are positively associated with synchronous CPM are discussed below. Due to a longer asymptomatic period, right-sided colon tumours are usually larger in diameter when they are diagnosed than left-sided colon tumours are. Larger neoplasms infiltrate the surface of the serosa over a larger area, which may lead to increased abscission of cancer cells into the peritoneal cavity. In addition, typical genetic differences between right-sided and left-sided colon tumours have been found, such as the BRAF status, and these genotypes may bring about a phenotype with a different probability of being associated with synchronous CPM (43). Several studies have shown that the mucinous histologic type has a poor prognostic impact, including a higher tendency for the development of peritoneal carcinomatosis and a lower response to oxaliplatin and irinotecan-based chemotherapy (44–46). A more advanced T stage is positively associated with the presence of peritoneal carcinomatosis, and the potential mechanism could be that peritoneal carcinomatosis is caused by serosal infiltration of the malignant tumour and subsequent abscission of cancer cells into the peritoneal cavity (47). Regarding peritoneal tumour spread, CA19-9 was shown to interact with E- and P-selectins that are expressed on human mesothelial and endothelial cells in the peritoneum (21, 48). Prokineticin1 (PROK1) is a known ligand of prokineticin receptor 2 (PROKR2) and transduces important molecular signals to induce physiological changes. The PROK1 protein has been identified as a vascular endothelial growth factor. Increased PROK1 expression is associated with angiogenesis involving haematogenous metastasis (31, 34). Several studies have analysed the association between PM and BRAF mutations. CRCs with BRAF mutations more frequently demonstrate adverse histologic features, such as lymphatic invasion, an increased mean number of lymph node metastases, perineural invasion, and a high amount of tumour budding (14, 49). In addition to direct invasion and haematogenous spread, peritoneal carcinomatosis can occur by lymphatic dissemination, which supports N1-2 being a risk factor (47, 50, 51).

There are some limitations in this study. First, non-English studies were excluded causing a language bias. Second, the risk associated with T4a vs. T4b stage was not analysed because there are no such detailed data. Finally, the number of included studies regarding CA19-9, PROK1/PROKR2, NRAS and PIK3CA was small, which may have limited its statistical power. We look forwards to conducting further studies.

## Conclusions

To our knowledge, this is the first meta-analysis to reveal the clinicopathological and molecular features of synchronous CPM. These evidence-based risk factors are conducive to strengthening the patient management and selecting the optimal therapeutic strategy.

## Data Availability Statement

The datasets used and/or analysed during the current study are available from the corresponding author on reasonable request.

## Author Contributions

HW designed the study. YZ, XQ, HMW, and HW performed the study selection and data extraction. YZ and RL performed the statistical analysis and data interpretation. YZ and XQ wrote and edited the manuscript. HL revised this manuscript. All authors read and approved the final draft.

## Funding

This study was sponsored by the National Natural Science Foundation of China (grant No. 82103084) and the Sun Yat-sen University Clinical Research 5010 Program (grant No. 2019021). The funding body had no role in the design of the study and collection, analysis, and interpretation of data and in writing the manuscript.

## Conflict of Interest

The authors declare that the research was conducted in the absence of any commercial or financial relationships that could be construed as a potential conflict of interest.

## Publisher’s Note

All claims expressed in this article are solely those of the authors and do not necessarily represent those of their affiliated organizations, or those of the publisher, the editors and the reviewers. Any product that may be evaluated in this article, or claim that may be made by its manufacturer, is not guaranteed or endorsed by the publisher.

## Abbreviations

PRISMA, Preferred Reporting Items of Systematic Reviews and Meta-analysis; PROSPERO, Prospective Register of Systematic Reviews; N-O score, Score of Newcastle–Ottawa Scale; OR, odds ratios; CI, confidence interval; CPM, colorectal peritoneal metastasis; CRC, colorectal cancer; PM, peritoneal metastases; pmCRC, peritoneal metastasis of colorectal cancer; CTGF, connective tissue growth factor; DDR2, discoidin domain receptor 2; VIM, vimentin; NMC, nonmucinous adenocarcinoma; MC, mucinous adenocarcinoma; SRCC, signet-ring cell carcinoma; PROK1, prokineticin1; PROKR2, prokineticin receptor2.
