# Supplementary material for: Risk Factors for Synchronous Peritoneal Metastases in Colorectal Cancer: A Systematic Review and Meta-Analysis
Source: Front Oncol. 2022 Jun 20;12:885504. doi: 10.3389/fonc.2022.885504 (PMC9251319; doi:10.3389/fonc.2022.885504)
Supplement: Supplementary file 1 [file DataSheet_1.docx]

# Supplementary Figure Captions

**Figure S1.** Funnel plot for estimation of publication bias. (a) Female. (b) T4. (c) N1-2. (d) Poorly differentiated grade.

**Figure S2.** Funnel plot for estimation of publication bias. (a) Right-sided colon. (b) Left-sided colon. (c) Rectum. (d) NMC.

**Figure S3.** Funnel plot for estimation of publication bias. (a) MC. (b) SRCC. (c) Serum CA19-9. (d) PROK1/PROKR2.

**Figure S4.** Funnel plot for estimation of publication bias. (a) BRAF. (b) KRAS. (c) NRAS. (d) PIK3CA.

**Figure S5.** Funnel plot for estimation of the MSI-H/dMMR’s publication bias.

**Figure S6.** Begg and Mazumdar rank correlation coefficients.

**Figure S1**


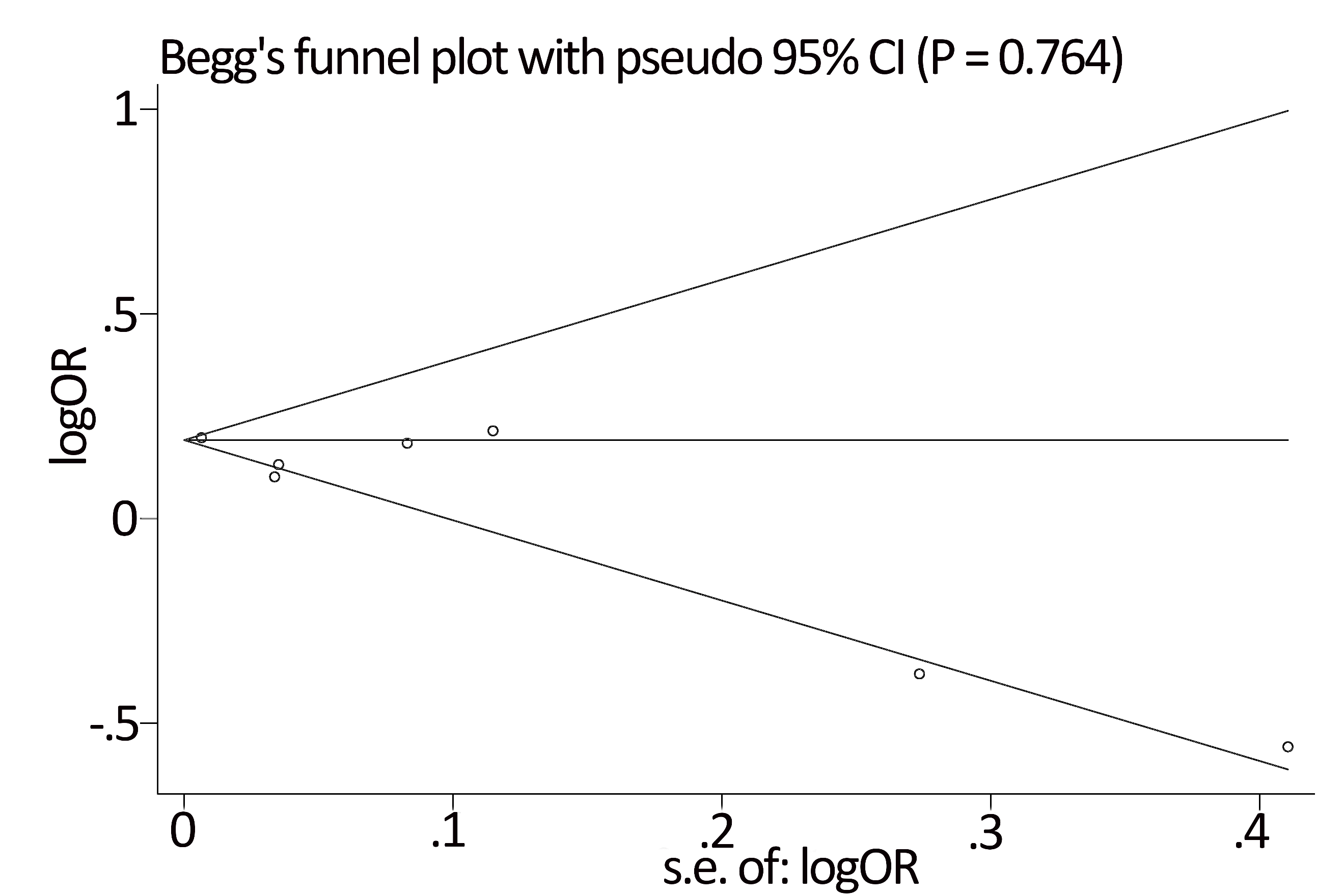

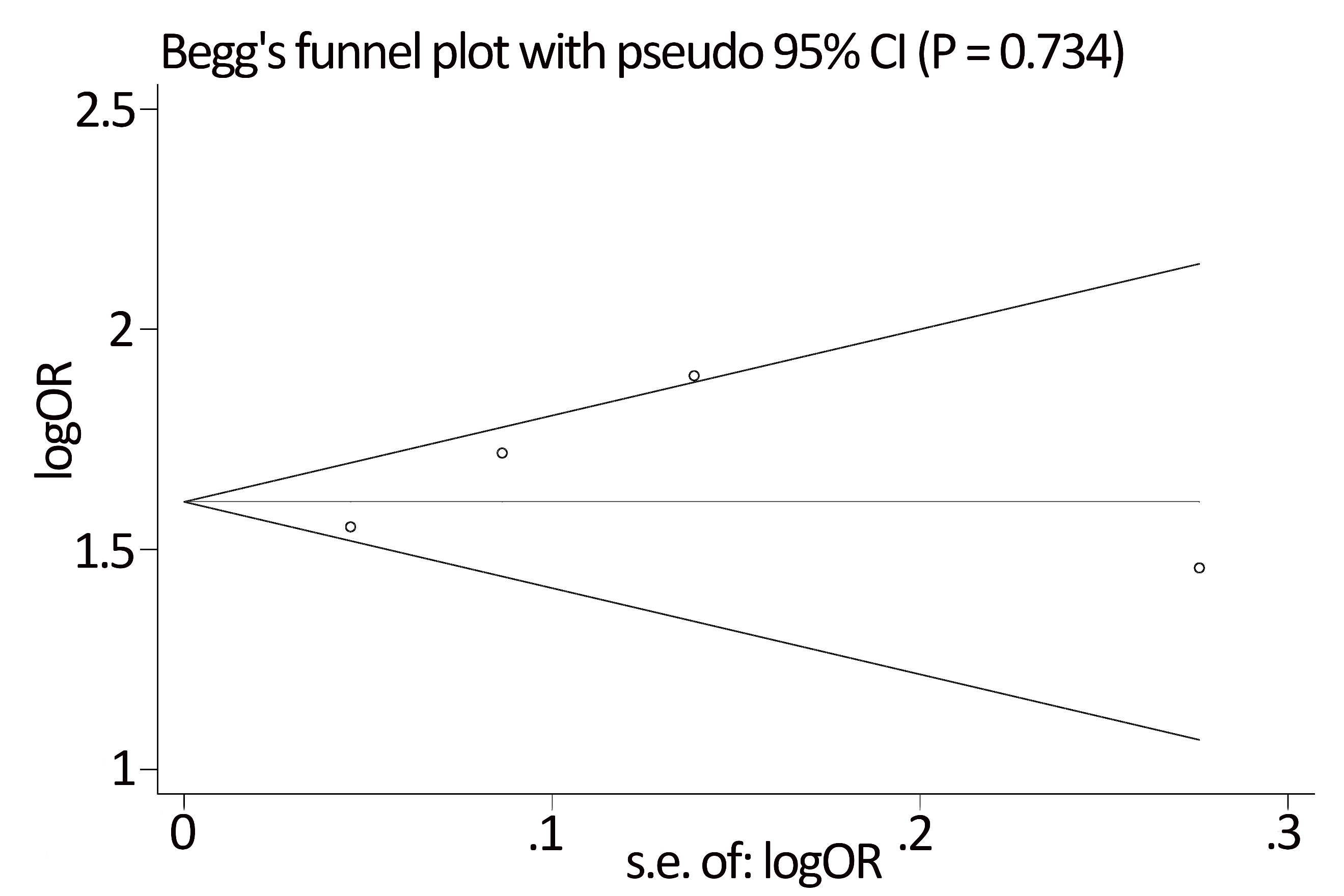


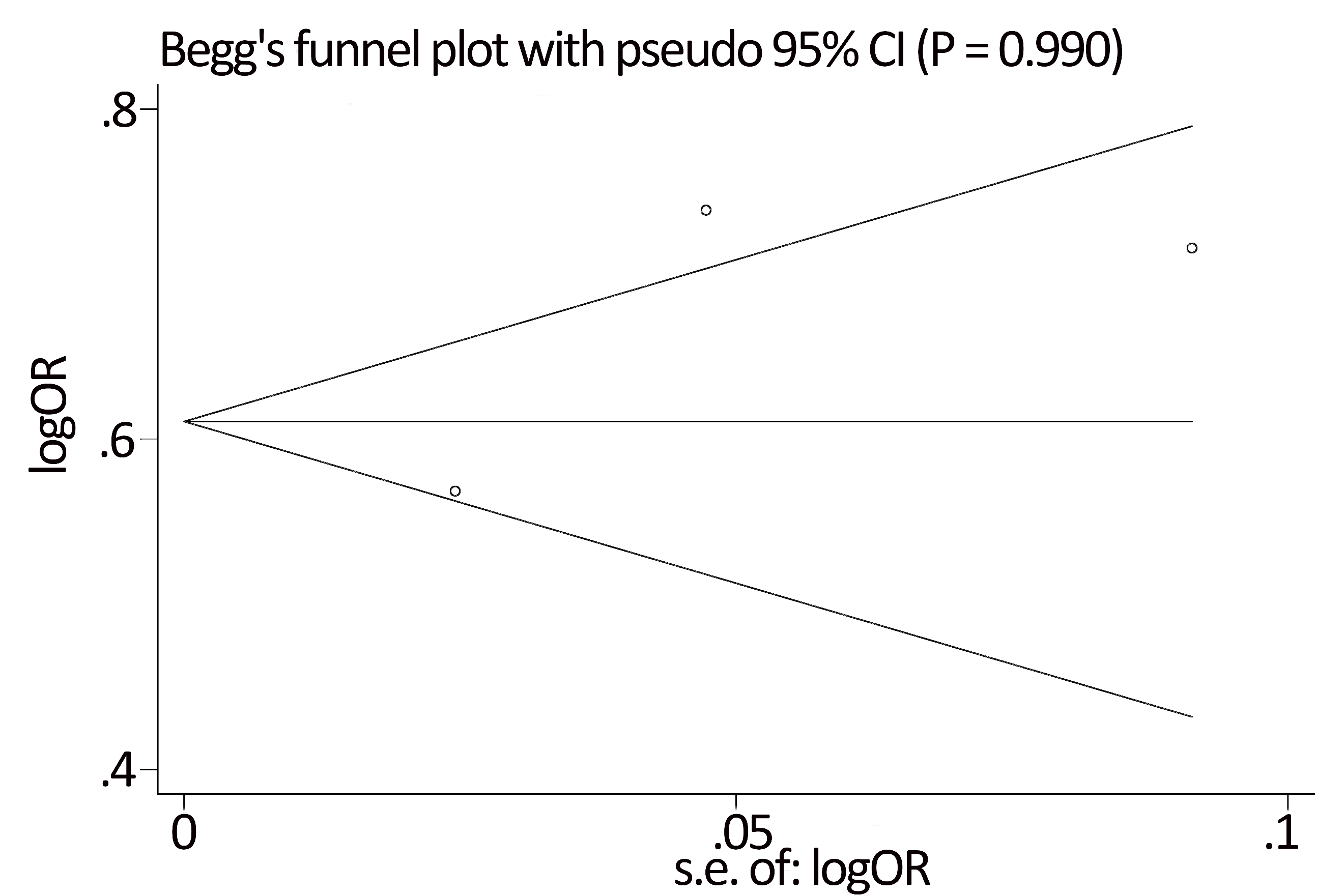

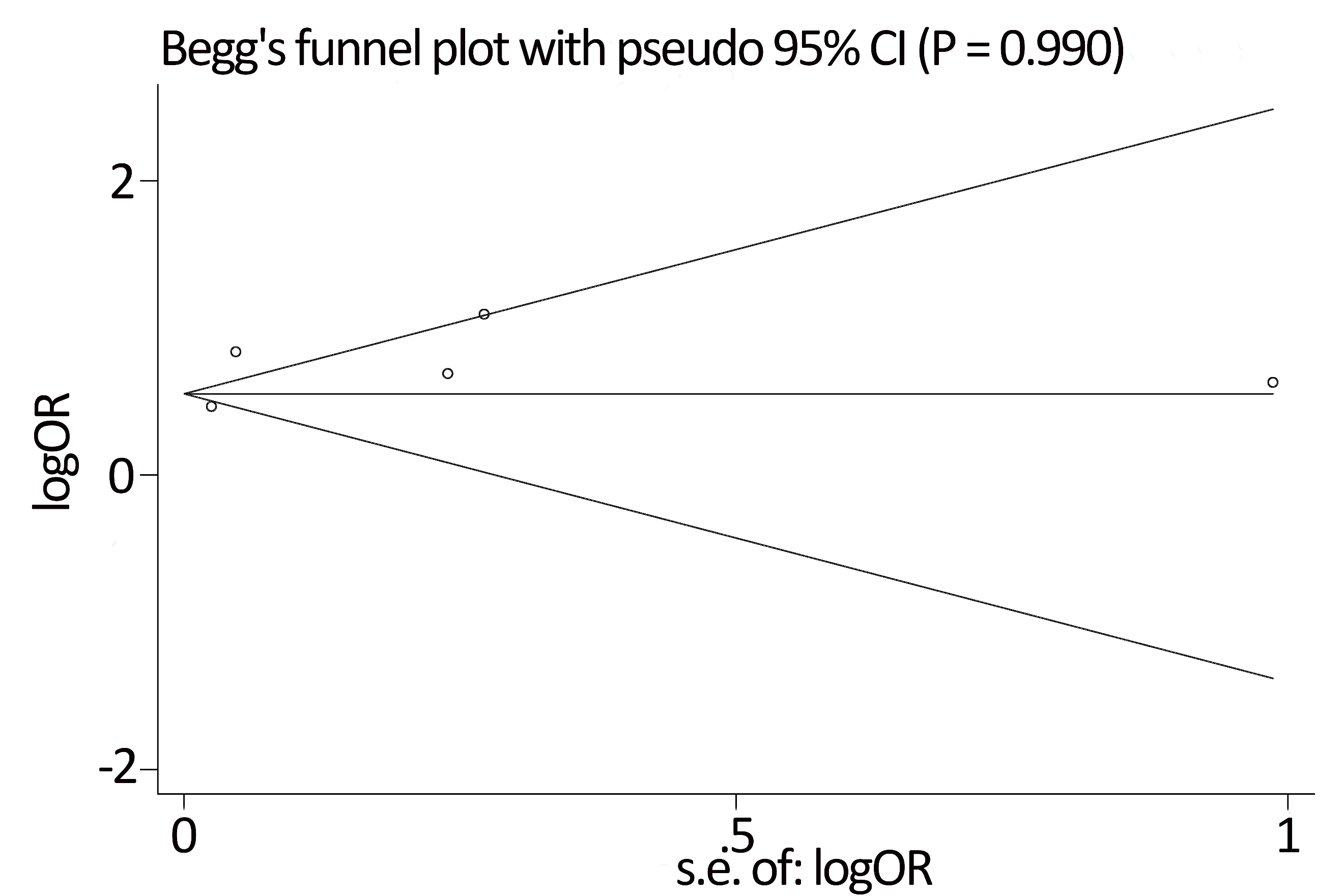


**a**

**b**

**c**

**d**

**Figure S2**


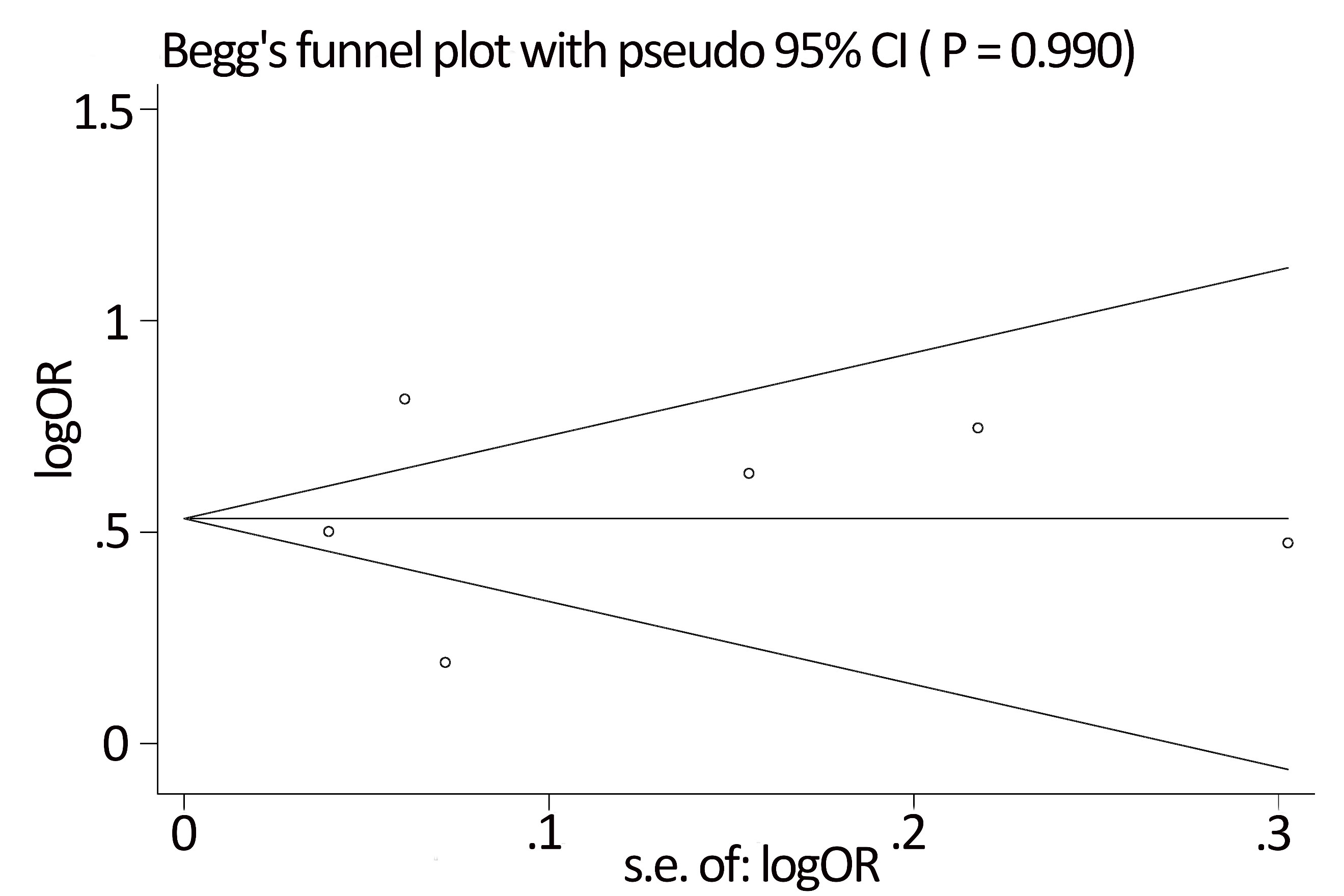

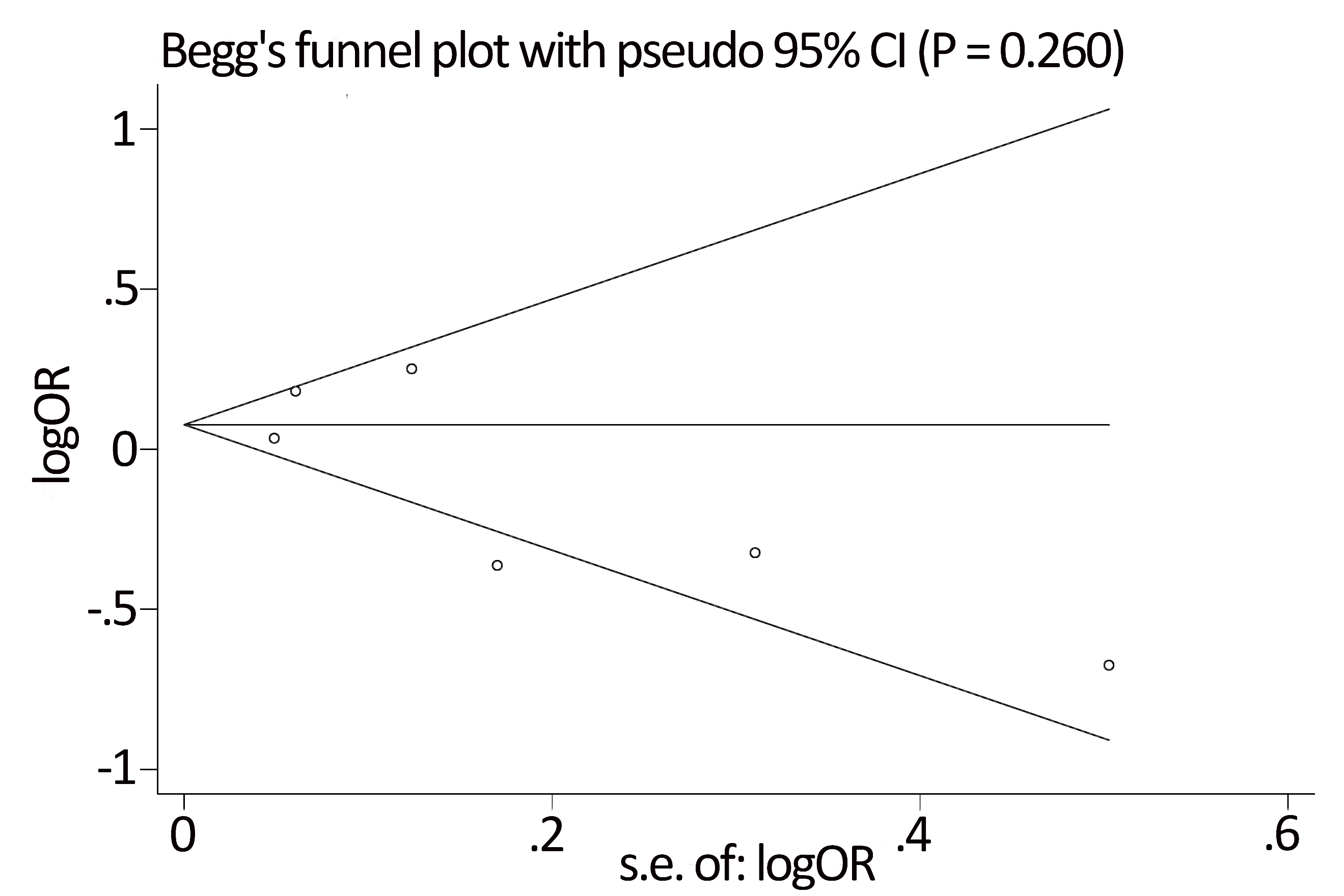


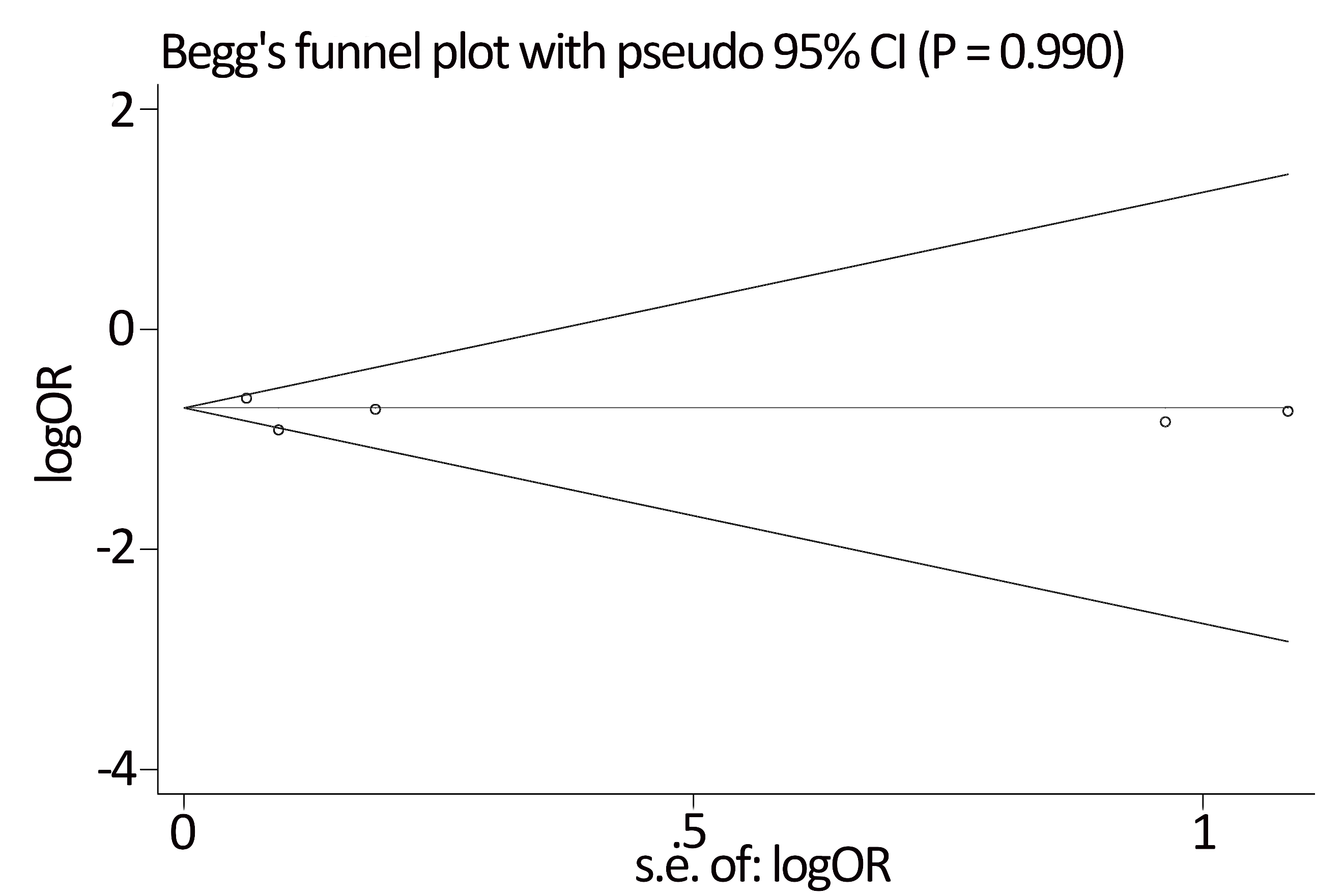

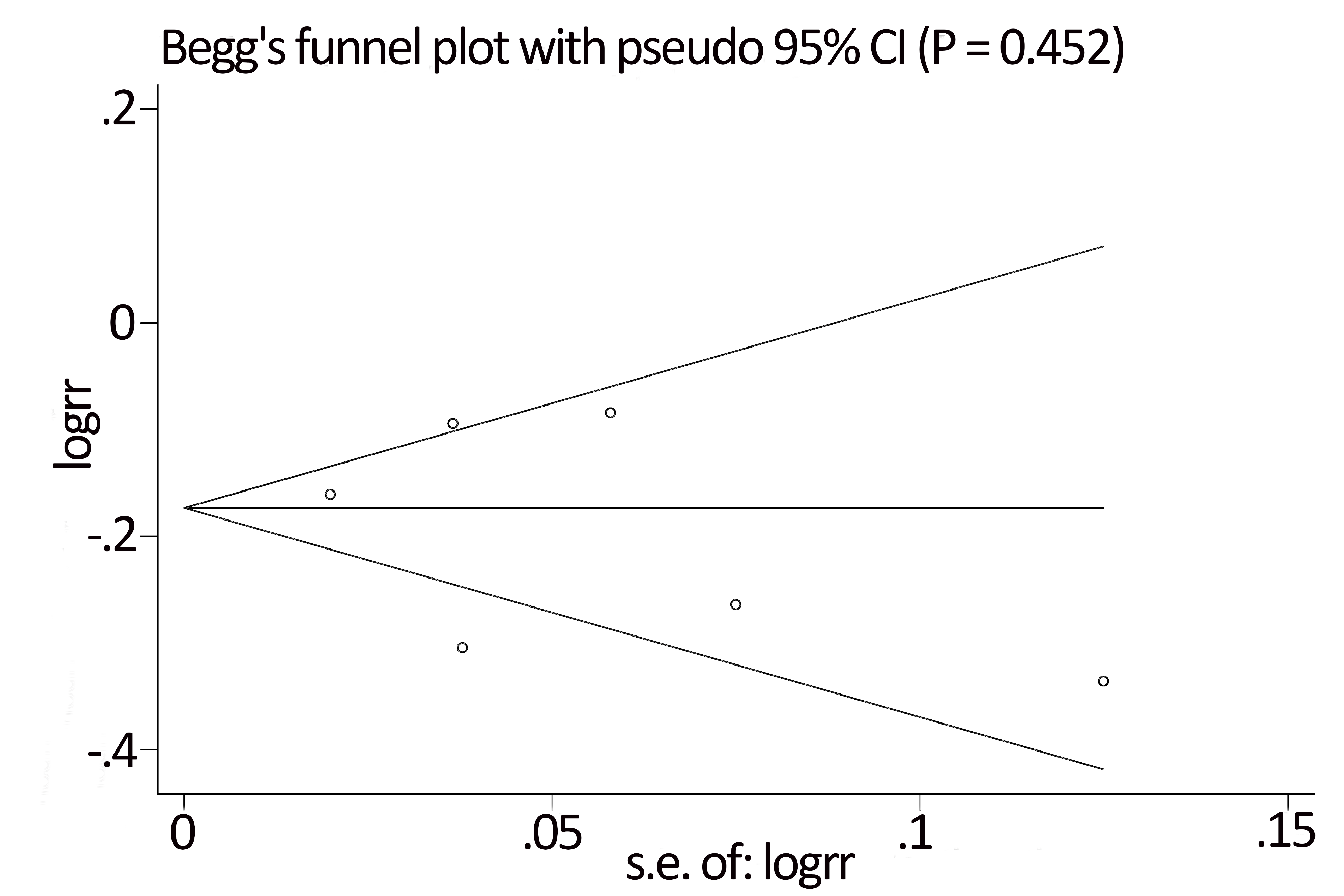


**a**

**b**

**c**

**d**


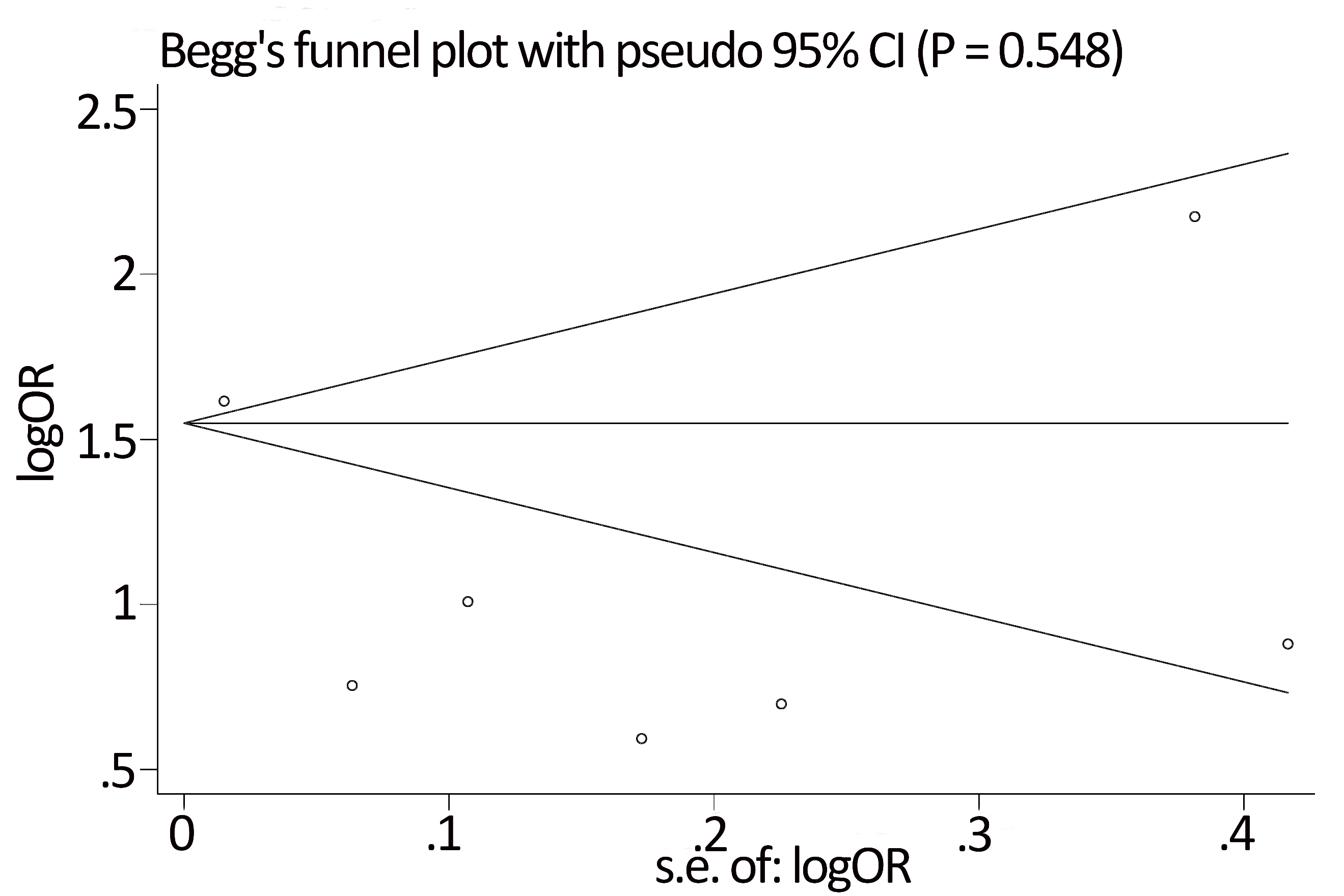

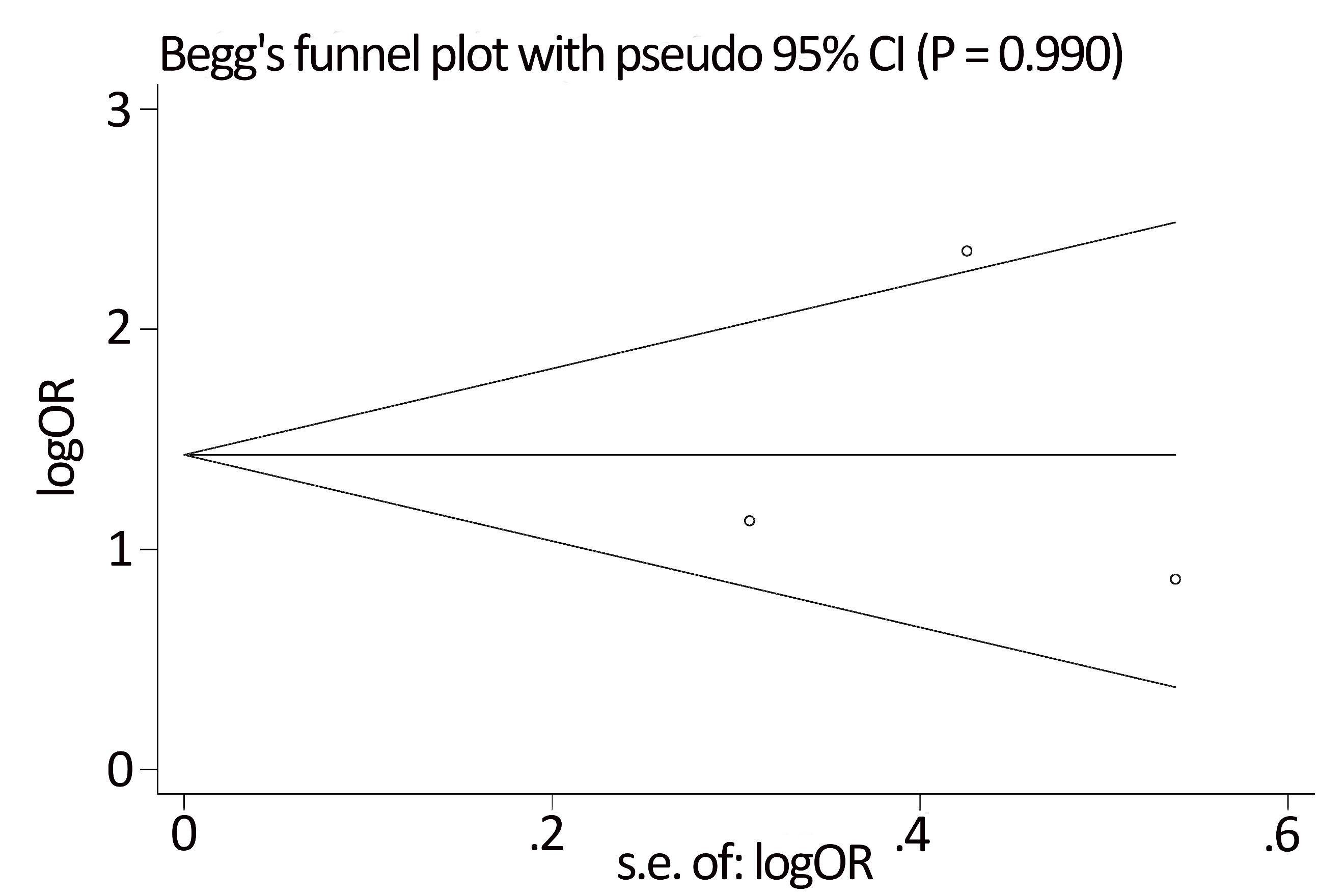


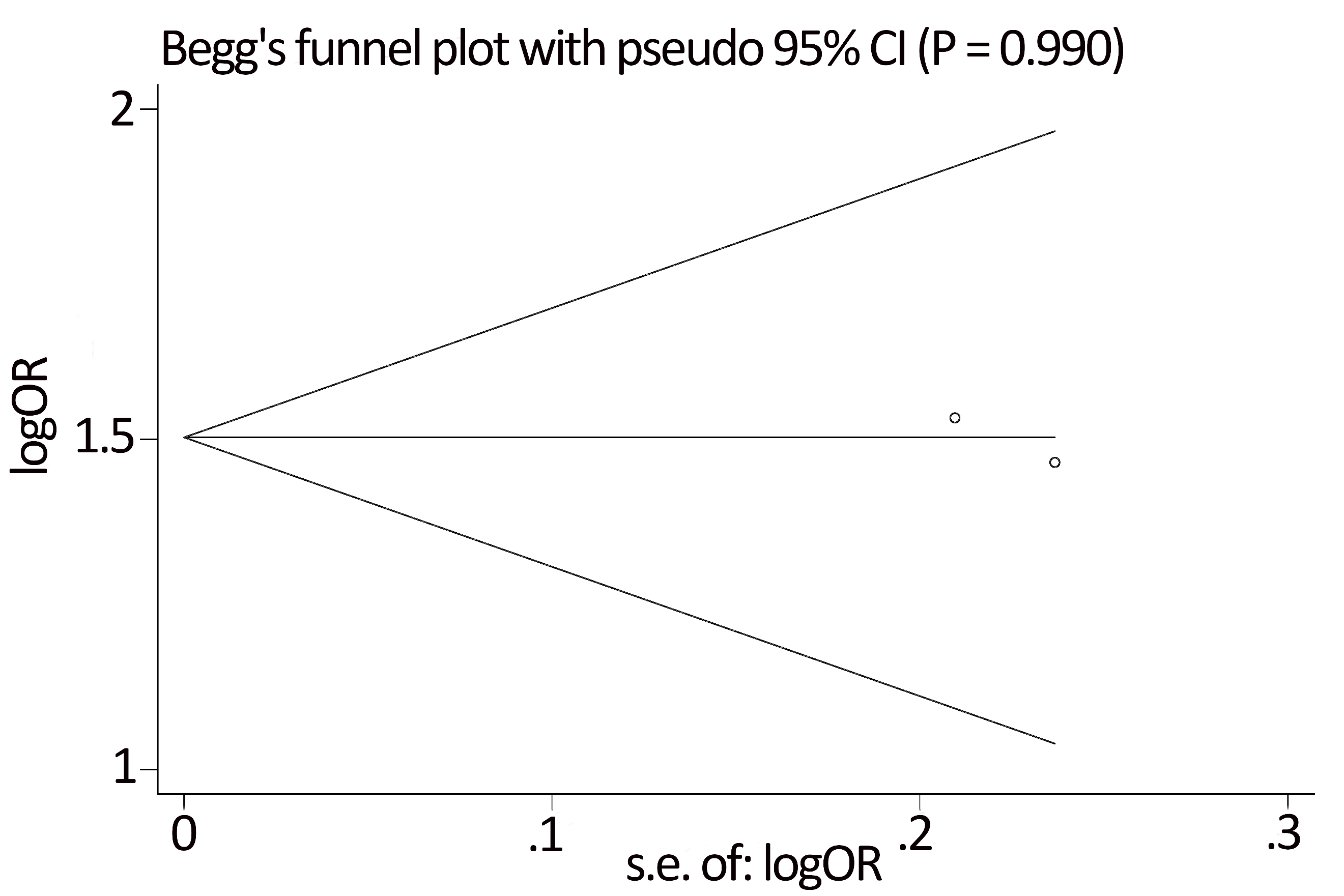

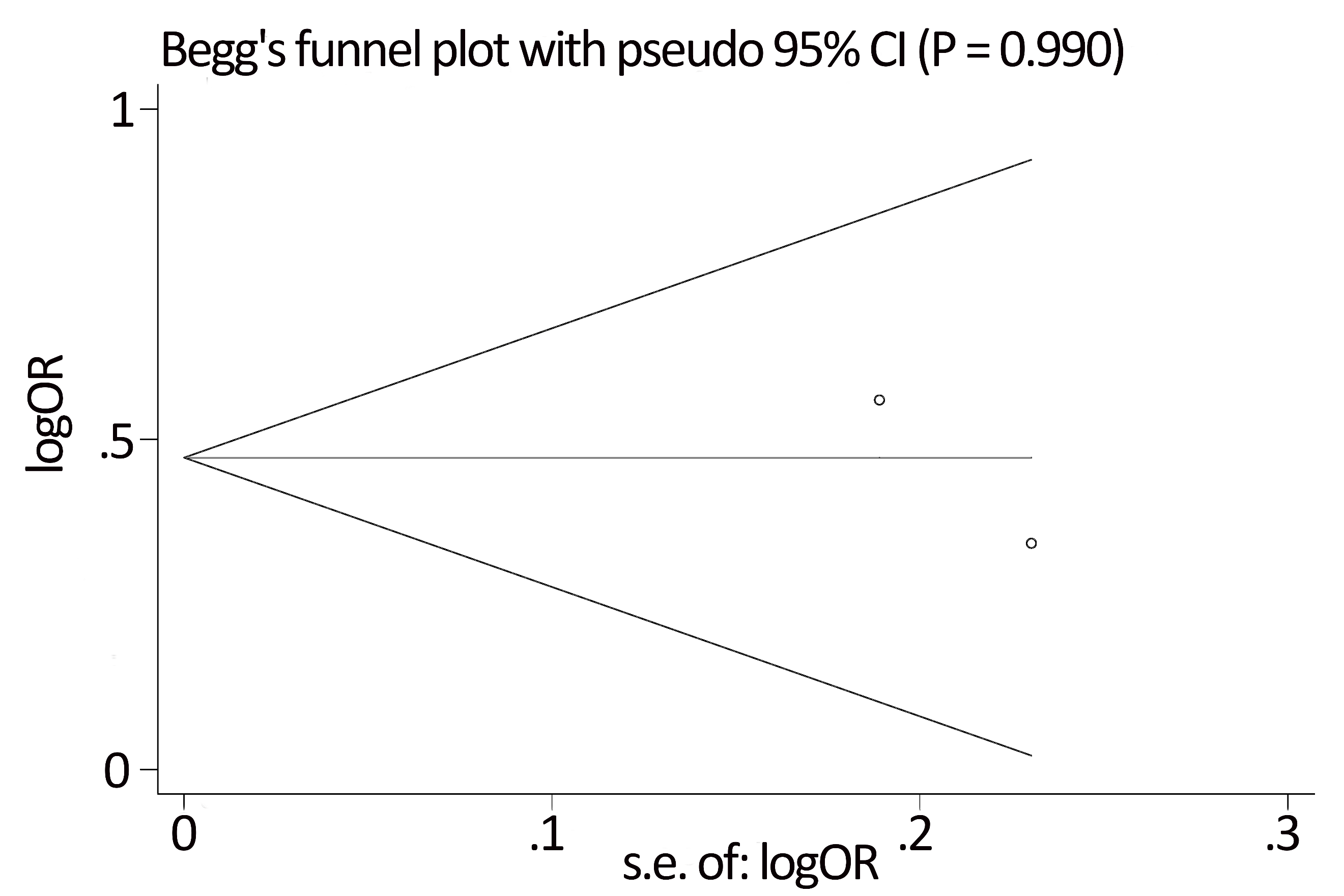


**a**

**b**

**c**

**d**

**Figure S3**

**Figure S4**


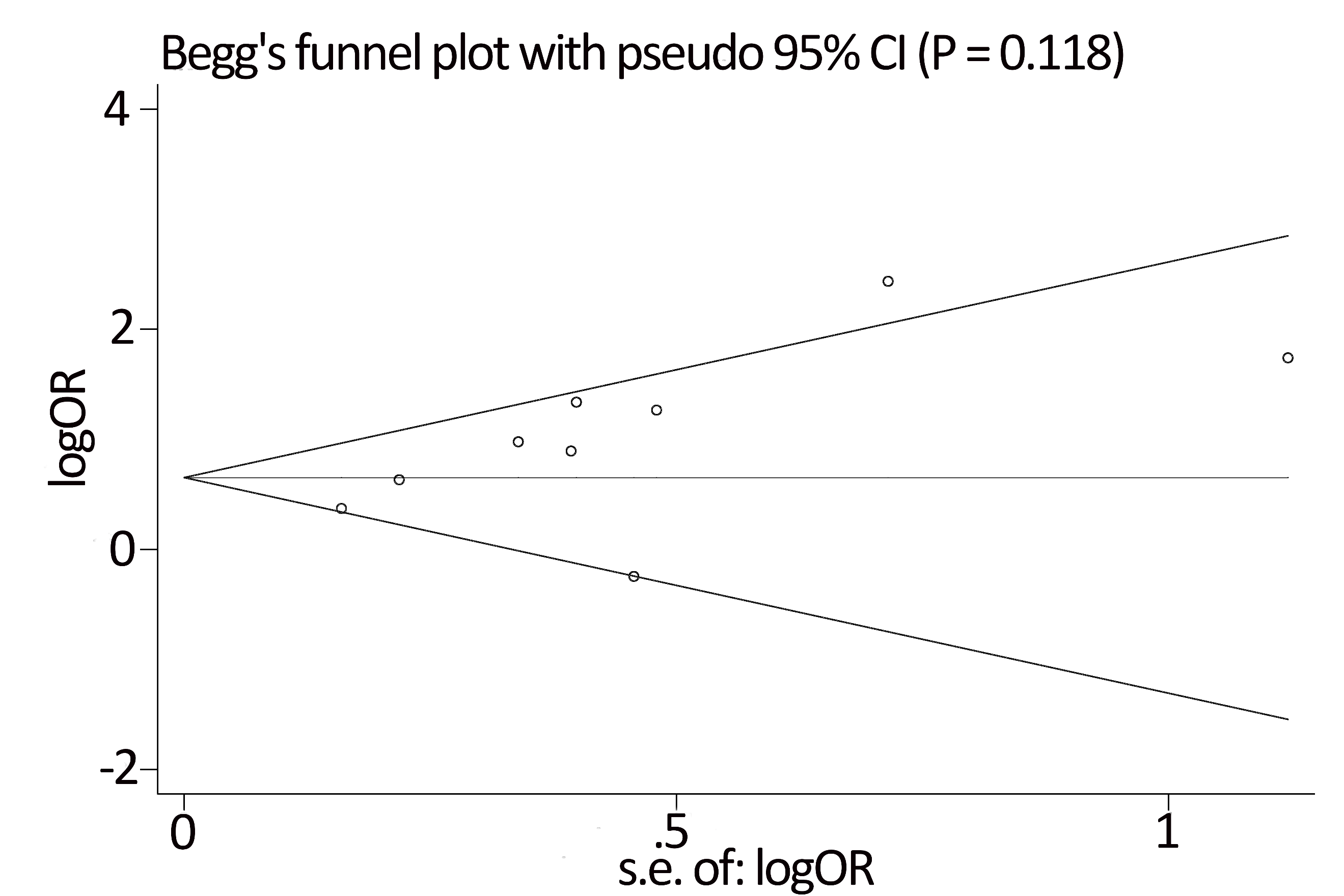

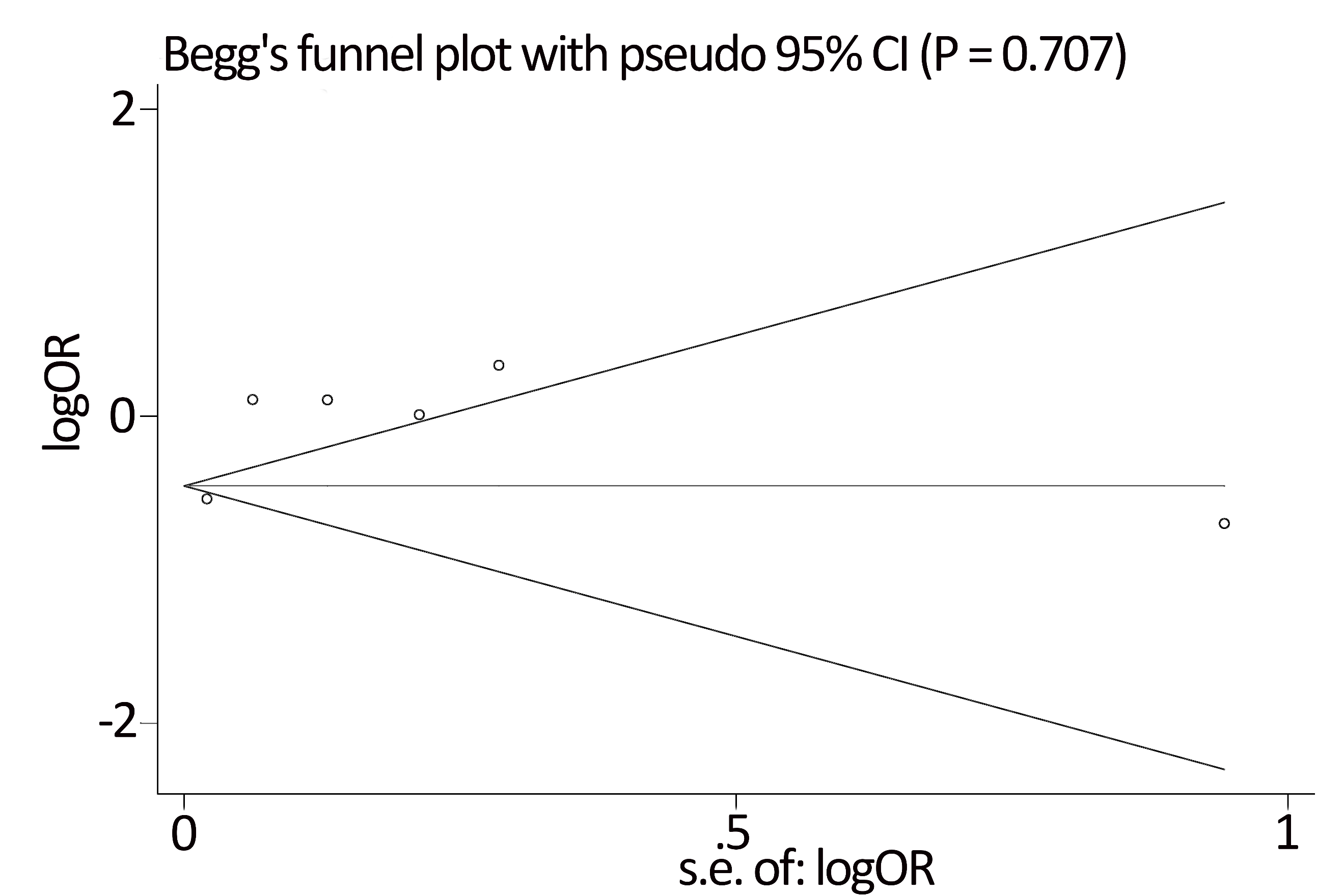


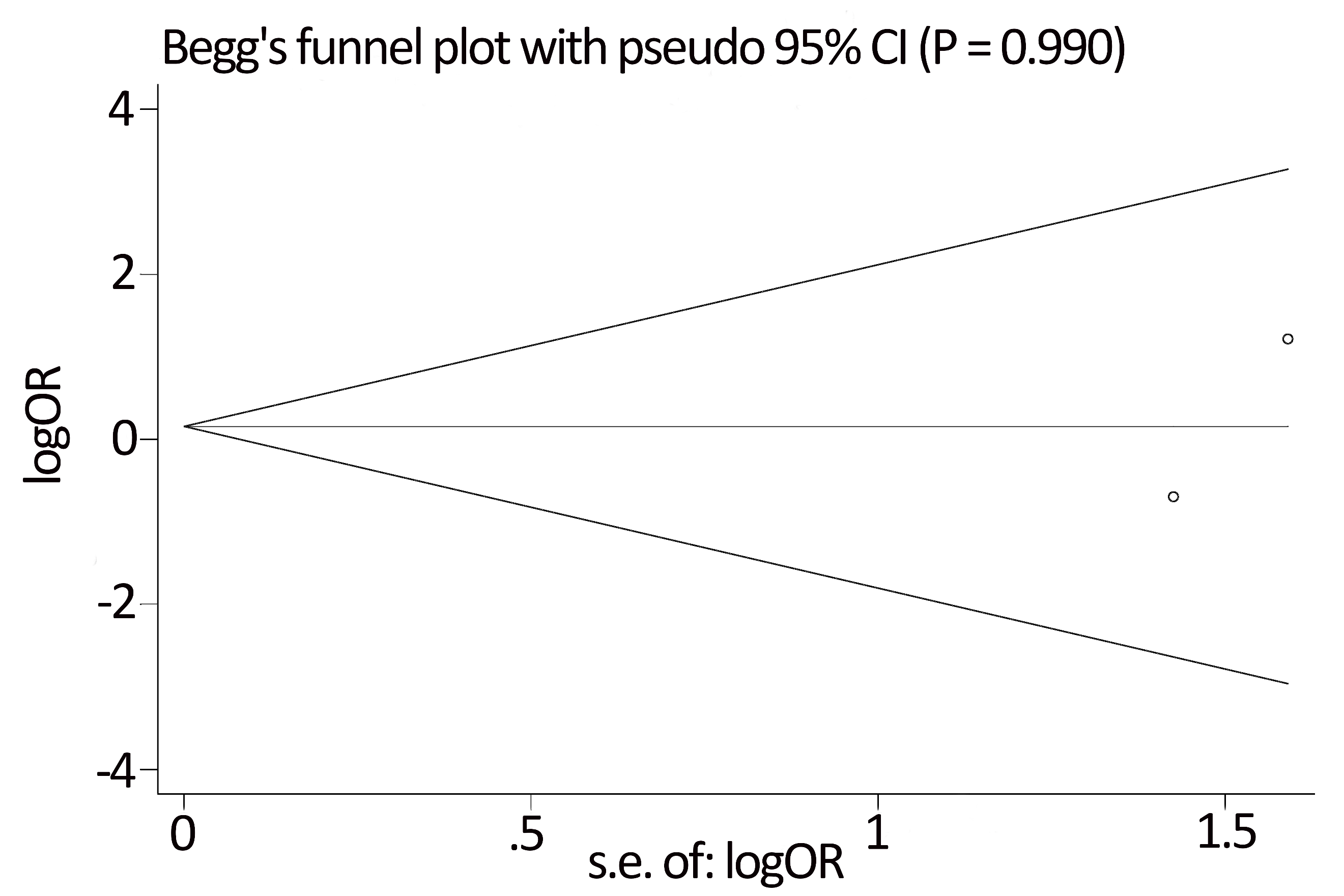

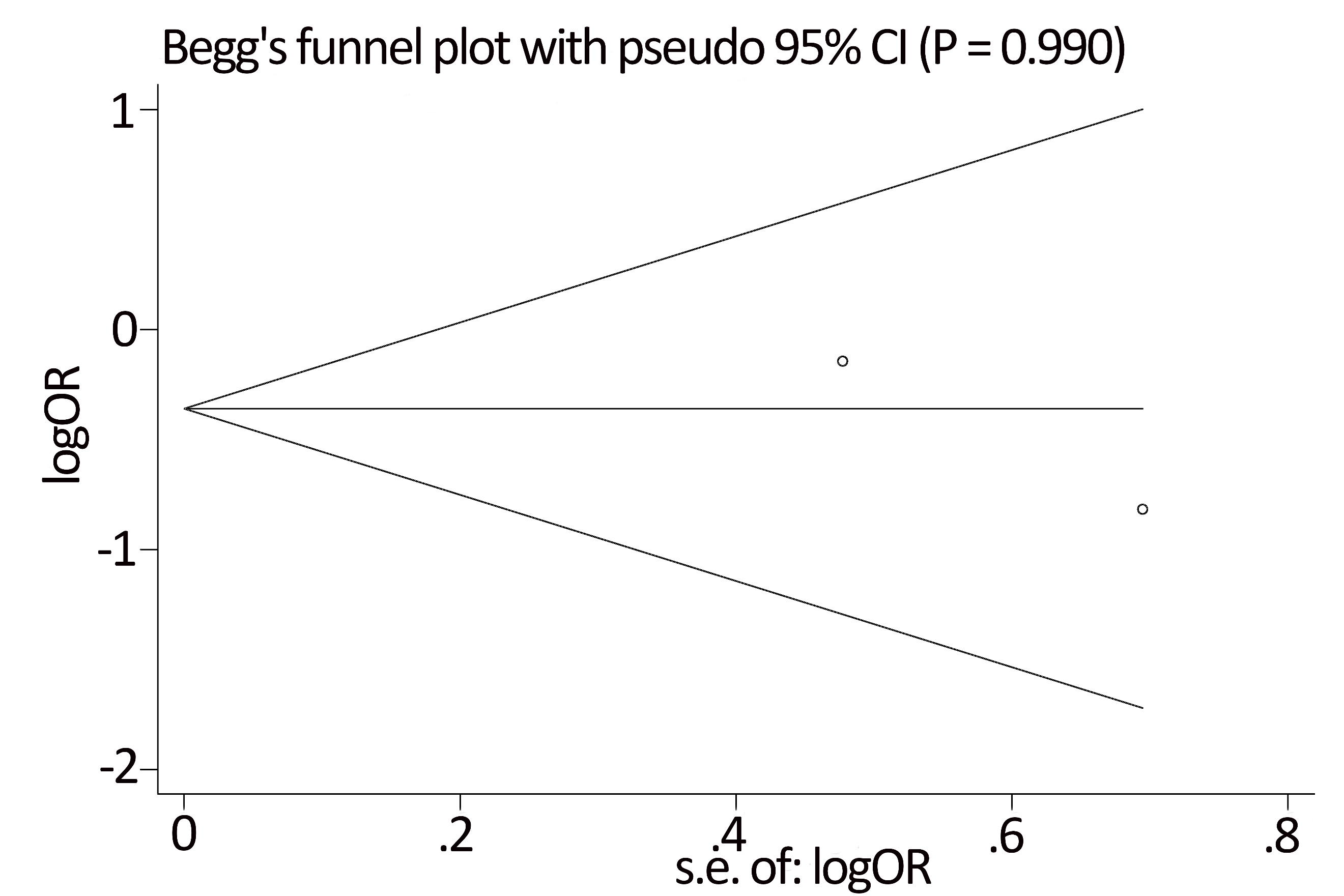


**a**

**b**

**c**

**d**

**Figure S5**


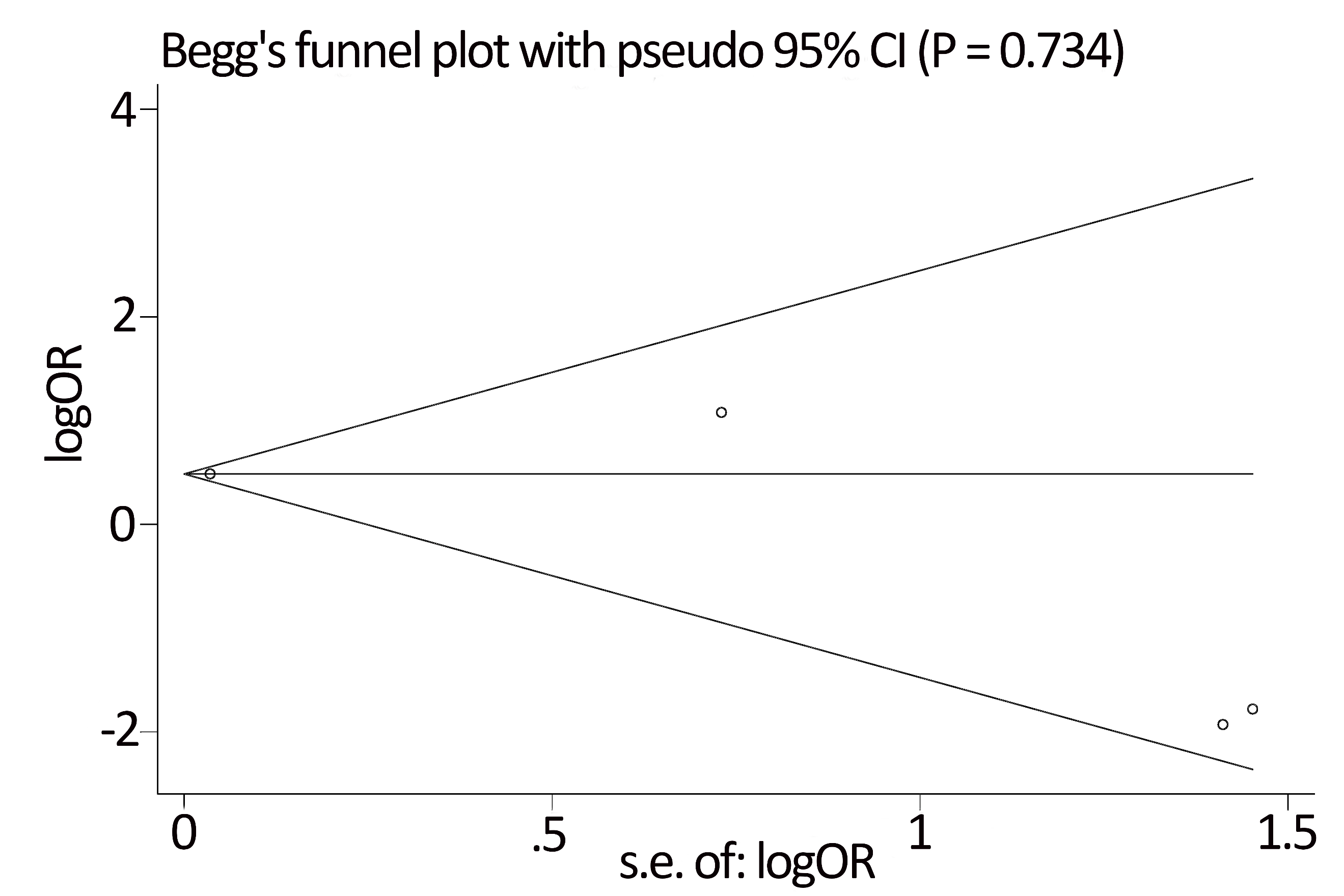


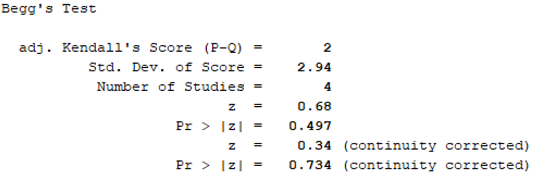

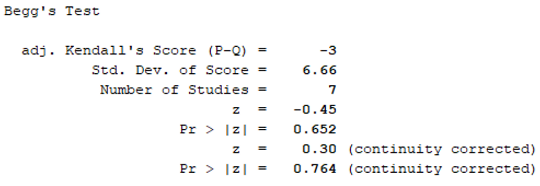
(1) Female (2) T4


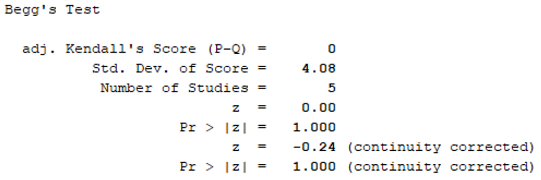
(3) N1-2 (4) Poorly differentiated grade


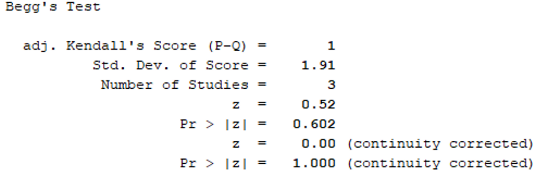


(5) Right-sided colon (6) Left-sided colon


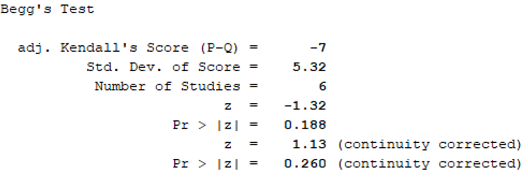

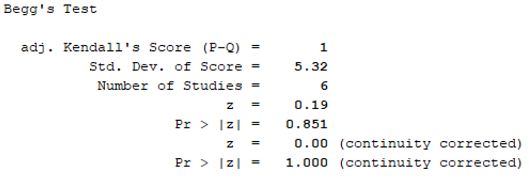


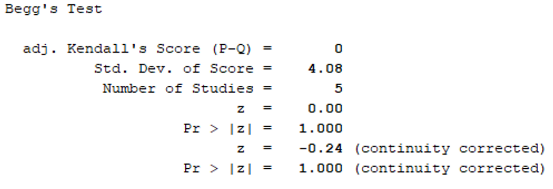
(7) Rectum (8) NMC


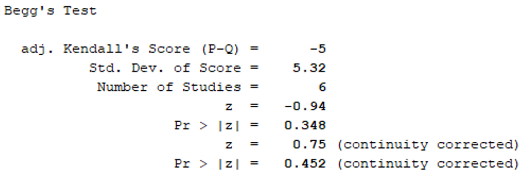


(9) MC (10) SRCC


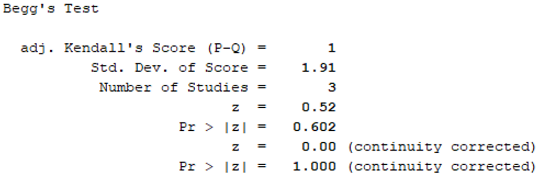

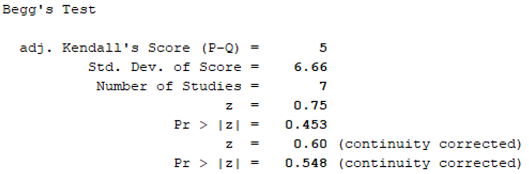


(11) Serum CA19-9 (12) PROK1/PROKR2


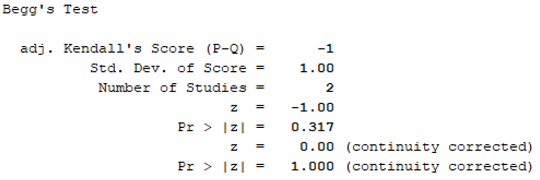

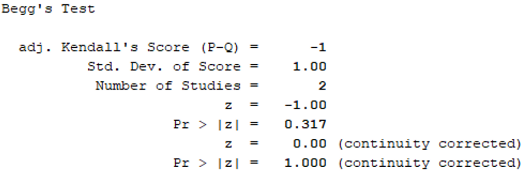


(13) BRAF (14) KRAS


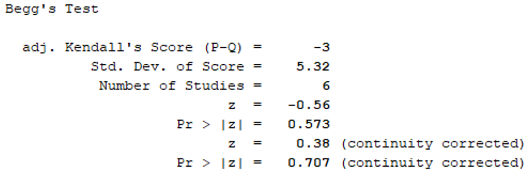

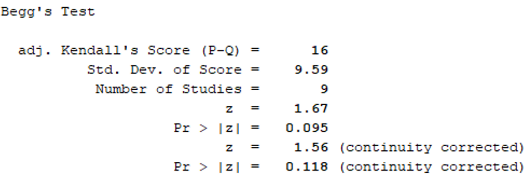


(15) NRAS (16) PIK3CA


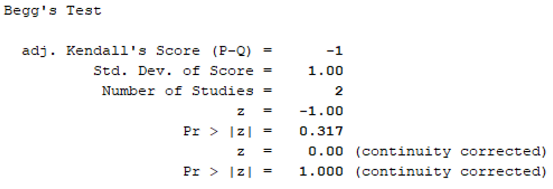

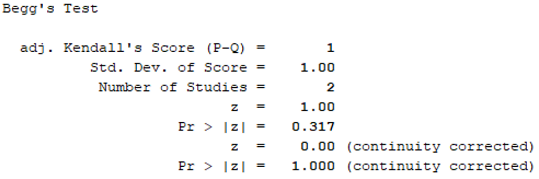


(17) MSI-H/dMMR


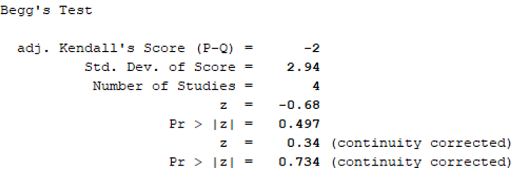


**Figure S6**
